# Glycine promotes longevity in *Caenorhabditis elegans* in a methionine cycle-dependent fashion

**DOI:** 10.1371/journal.pgen.1007633

**Published:** 2019-03-07

**Authors:** Yasmine J. Liu, Georges E. Janssens, Rebecca L. McIntyre, Marte Molenaars, Rashmi Kamble, Arwen W. Gao, Aldo Jongejan, Michel van Weeghel, Alyson W. MacInnes, Riekelt H. Houtkooper

**Affiliations:** 1 Laboratory Genetic Metabolic Diseases, Amsterdam Gastroenterology and Metabolism, Amsterdam UMC, University of Amsterdam, Meibergdreef 9, Amsterdam, The Netherlands; 2 Bioinformatics Laboratory, Department of Clinical Epidemiology, Biostatistics and Bioinformatics, Amsterdam UMC, University of Amsterdam, Meibergdreef 9, AZ Amsterdam, the Netherlands; Princeton, UNITED STATES

## Abstract

The deregulation of metabolism is a hallmark of aging. As such, changes in the expression of metabolic genes and the profiles of amino acid levels are features associated with aging animals. We previously reported that the levels of most amino acids decline with age in *Caenorhabditis elegans (C*. *elegans)*. Glycine, in contrast, substantially accumulates in aging *C*. *elegans*. In this study we show that this is coupled to a decrease in gene expression of enzymes important for glycine catabolism. We further show that supplementation of glycine significantly prolongs *C*. *elegans* lifespan, and early adulthood is important for its salutary effects. Moreover, supplementation of glycine ameliorates specific transcriptional changes that are associated with aging. Glycine feeds into the methionine cycle. We find that mutations in components of this cycle, methionine synthase (*metr-1*) and S-adenosylmethionine synthetase (*sams-1*), completely abrogate glycine-induced lifespan extension. Strikingly, the beneficial effects of glycine supplementation are conserved when we supplement with serine, which also feeds into the methionine cycle. RNA-sequencing reveals a similar transcriptional landscape in serine- and glycine-supplemented worms both demarked by widespread gene repression. Taken together, these data uncover a novel role of glycine in the deceleration of aging through its function in the methionine cycle.

## Author summary

There is a growing number of studies showing that amino acids function as signal metabolites that influence aging and health. Although contemporary -OMICs studies have uncovered various associations between metabolite levels and aging, in many cases the directionality of the relationships is unclear. In a recent metabolomics study, we found that glycine accumulates in aged *C*. *elegans* while other amino acids decrease. The present study shows that glycine supplementation increases lifespan and drives a genome-wide inhibition effect on *C*. *elegans* gene expression. Glycine as a one-carbon donor fuels the methyl pool of one-carbon metabolism composed of the folate and methionine cycles. We find that the glycine-mediated longevity effect is fully dependent on the methionine cycle, and that all of our observations are conserved with supplementation of the other one-carbon amino acid, serine. These results provide a novel role for glycine as a promoter of longevity and bring new insight into the role of one-carbon amino acids in the regulation of aging that may ultimately be beneficial for humans.

## Introduction

Aging is characterized by a progressive deterioration of the functional capacity of tissues and organs. Pioneering studies in the nematode *C*. *elegans* have identified longevity-associated genes and provided us with great insights into the plasticity of aging [1–3]. In the last few decades, genetic and nutritional interventions have been employed in multiple organisms including *Saccharomyces cerevisiae*, *C*. *elegans*, *Drosophila melanogaster*, rodents, and more recently fish [4–6]. These models have set the stage for characterizing the genetic basis of physiological aging and for developing efficient strategies to control the rate of aging.

To date, metabolic pathways including the mTOR, insulin/IGF-1, and AMP-activated protein kinase (AMPK) signaling pathways have emerged as playing a critical role in aging [reviewed in [7]]. Several studies demonstrate that the levels of specific amino acids effectively influence lifespan by affecting these pathways. For example, the branched-chain amino acids valine, leucine, and isoleucine when administered to *C*. *elegans* can function as signaling metabolites that mediate a mTOR-dependent neuronal-endocrine signal that in turn promotes a longer lifespan [8]. Moreover, inhibition of threonine and tryptophan degradation also contributes to lifespan extension by enhancing protein homeostasis in *C*. *elegans* [9,10]. Additionally, restriction of methionine extends the lifespan of flies in a mTOR-dependent manner [11]. However, supplementation of other amino acids such as methionine, serine, glycine, histidine, arginine, and lysine have been shown to promote lifespan in *C*. *elegans* by mechanisms that are to date not known [12].

In addition to the mTOR signaling pathway, alterations in one-carbon metabolism involving the folate and methionine cycles couple amino acid metabolism to the regulation of human health and disease [13]. Glycine, as one of the input amino acids that feeds into one-carbon metabolism, provides a single carbon unit to the folate cycle to yield a variety of one-carbon bound tetrahydrofolates (THFs) [14]. These function as coenzymes in methylation reactions including the production of methionine through methionine synthase (METR-1 in *C*. *elegans*) as well as the universal methyl donor, S-adenosylmethionine (SAMe) through S-adenosyl methionine synthetase (SAMS-1 in *C*. *elegans*) [14]. These output metabolites of one-carbon metabolism support a range of biological functions [14]. In *C*. *elegans*, mutations in the metabolic gene *sams-1* and the levels of SAMe and S-adenosylhomocysteine (SAH) have been implicated in the regulation of aging [15,16]. Although the underlying mechanism of how SAMe/SAH status influences aging needs further investigation, studies *in vivo* have provided evidences that the level of SAMe couples with the trimethylation status of lysine 4 on histone H3 (H3K4me3) and affects gene regulation [17]. Another study in mouse pluripotent stem cells demonstrates that threonine catabolism contributes one carbon to SAMe synthesis and histone methylation through glycine cleavage pathway [18]. Of particular note, several histone methyl-transferases and de-methyltransferases in *C*. *elegans* have been identified as longevity regulators [19–21]. Taken together, these studies all suggest that altering one-carbon metabolism is a mechanism that controls the aging process.

We recently showed that glycine accumulates with age in a large scale metabolomics study profiling levels of fatty acids, amino acids, and phospholipids across the lifespan of *C*. *elegans*, [22]. Another study in human fibroblasts suggested that epigenetic suppression of two nuclear-coded genes, glycine C-acetyltransferase (*GCAT*) and serine hydroxymethyltransferase 2 (*SHMT2*) which are both involved in glycine synthesis in mitochondria, was partly responsible for aging-associated mitochondrial respiration defects [23]. This study went on to report that glycine treatment rejuvenated the respiration capacity of fibroblasts derived from elderly individuals [23]. However, to date the role of glycine has not been systematically defined in animal models of longevity. In this study, we build upon our previous observations that suggest glycine accumulation in aging animals may play a unique and as-of-yet unexplored role in the regulation of eukaryote lifespan.

## Results

### Genes coding for glycine degradation enzymes decrease with age in *C*. *elegans* as glycine accumulates

We previously measured amino acid levels throughout the life of *C*. *elegans*, including four larval phases (L1-L4) and ten days of adulthood from young worms to aged ones (days 1–10) [22]. We reported that the concentrations of most amino acids peaked at the later larval stage or early adult phase and then began declining at different adult stages, reaching low levels by the latest stages of the animals’ life [22]. One stark exception was glycine, which continued to accumulate in aged worms [22].

**Fig 1:**
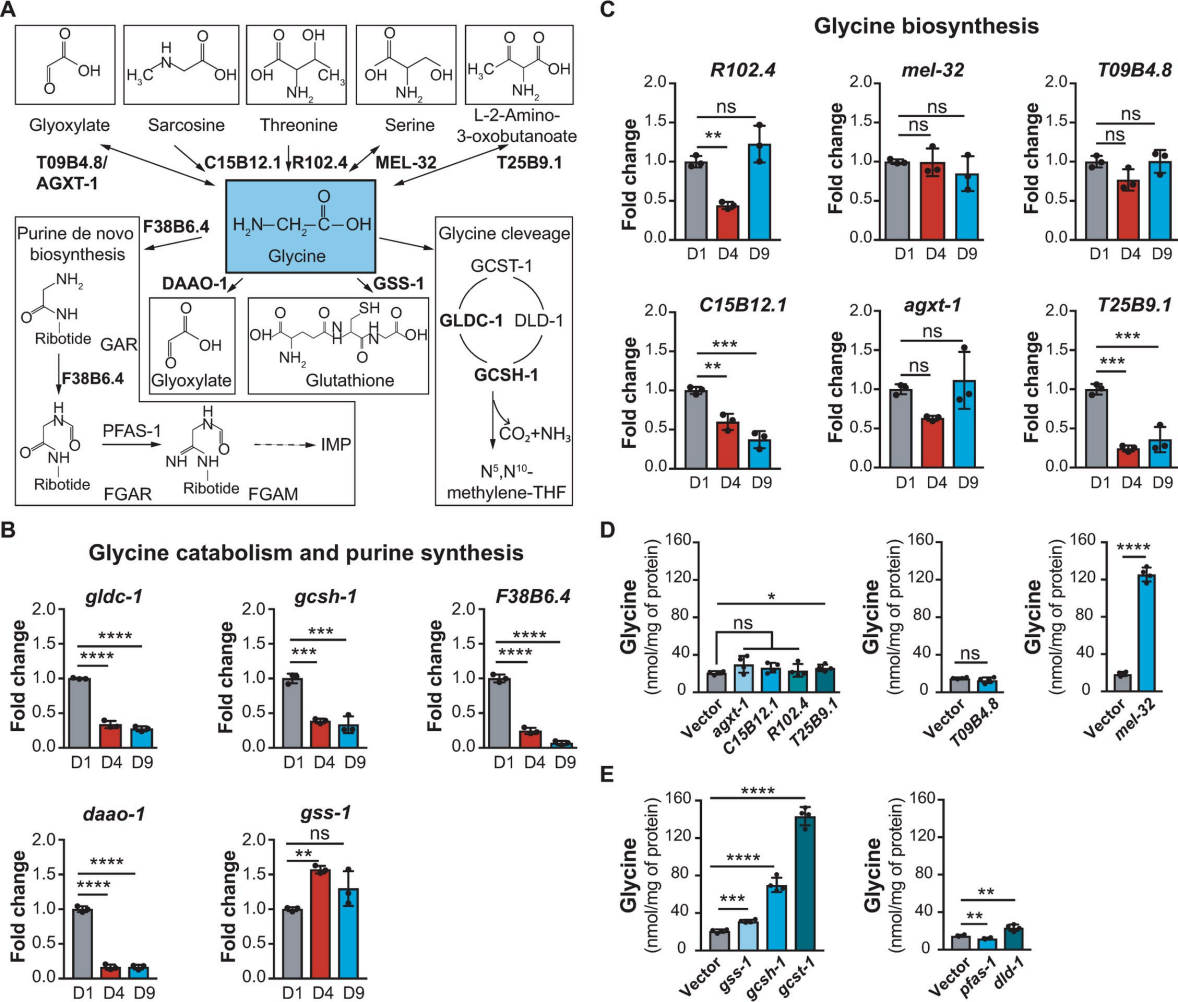
The abundance of glycine increases with age in *C*. *elegans*. **(A)** Glycine metabolic pathways in *C*. *elegans* with genes used in RT-qPCR. This diagram was constructed according to “glycine, serine and threonine metabolism” (cel00260) in the KEGG pathway database and the metabolic network of glycine in WormFlux [64,65]. **(B)** Transcript levels of genes involved in glycine catabolism and *de novo* purine synthesis showing that *gldc-1*, *gcsh-1*, *F38B6*.*4*, and *daao-1* decrease markedly in aged worms (D9), while the expression of *gss-1* peaks at D4 and subsequently decreases at D9 to the same level as that at D1. Worms were cultured on alive *E*. *coli* OP50. Results are shown relative to transcript levels in D1 adult worms. Bar graphs are expressed as mean ± SD with three biological replicates; Significance was calculated using one-way ANOVA; **p < 0.01; ***p < 0.001; ****p < 0.0001; ns, not significant. **(C)** Transcript levels of genes involved in glycine biosynthesis measured by RT-qPCR showing that the expressions of most genes assessed including *R102*.*4*, *mel-32*, *T09B4*.*8*, and *agxt-1* are quite stable throughout the life stages of worms including D1, D4, and D9. Worms were cultured on alive *E*. *coli* OP50. Results are shown relative to transcript levels in D1 adult worms. Bar graphs are expressed as mean ± SD with three biological replicates; Significance was calculated using one-way ANOVA; **p < 0.01; ***p < 0.001; ns, not significant. **(D)** Glycine levels measured by UPLC-MS/MS in worms fed *E*. *coli* HT115 expressing dsRNA against genes in glycine biosynthesis pathways including *T09B4*.*8*, *agxt-1*, *C15B12*.*1*, *R102*.*4*, *T25B9*.*1* and *mel-32*. RNAi depletion of *mel-32* leads to a marked increase of the endogenous level of glycine. In contrast, RNAi of other genes such as *T09B4*.*8*, *agxt-1*, *C15B12*.*1*, *R102*.*4* and *T25B9*.*1* has limited effects on the endogenous level of glycine. Worms were fed RNAi bacteria from the time of hatching and collected for amino acids extraction at D1. Bar graphs are expressed as mean ± SD with four biological replicates; Significance was calculated using Student’s *t*-test; *p < 0.05, ****p < 0.0001; ns, not significant. **(E)** Glycine levels measured by UPLC-MS/MS in worms fed *E*. *coli* HT115 expressing dsRNA targeting genes in glycine catabolism and purine synthesis including *gss-1*, *gcsh-1*, *gcst-1*, *pfas-1*, and *dld-1*. RNAi depletions of genes in glycine catabolism including *gss-1*, *gcsh-1*, *gcst-1*, and *dld-1* significantly increase the endogenous level of glycine to different extents. Worms were fed RNAi bacteria from the time of hatching and collected for amino acids extraction at D1. Bar graphs are expressed as mean ± SD with four biological replicates; Significance was calculated using Student’s *t*-test; **p < 0.01; ***p < 0.001; ****p < 0.0001.

To determine if the accumulation of glycine with age is due to increased synthesis or reduced degradation, we measured the expression levels of genes directly involved in glycine metabolism in worms collected at different ages (Fig 1A). Specifically, we observed that the expression levels of most genes in glycine degradation and consumption pathways including glycine decarboxylase (*gldc-1*), glycine cleavage system H protein (*gcsh-1*), phosphoribosylamine-glycine ligase (*F38B6*.*4*), and D-amino acid oxidase (*daao-1*) were dramatically lower at day 9 of adulthood (D9) (Fig 1B). In contrast, the expression levels of most genes involved in glycine synthesis including threonine aldolase (*R102*.*4*), serine hydroxymethyltransferase (*mel-32*), alanine-glyoxylate aminotransferase 2 (*T09B4*.*8*), and alanine-glyoxylate amino transferase (*agxt-1*) remained unchanged in aged worms (Fig 1C). These data suggest that the accumulation of glycine observed in aged worms is predominantly due to a reduction in the expression of genes required for its degradation.

To gain a better understanding of the significance of glycine accumulation in aged animals, we next asked whether this phenomenon is prevalent among long-lived worms such as *daf-2(e1370)* and *eat-2(ad465)*, the *C*. *elegans* models of impaired insulin signalling [2] and dietary restriction [24], respectively. To characterize the changes in the levels of glycine during aging in the *daf-2(e1370)* and *eat-2(ad465)* mutant lines, we measured glycine levels in long-lived worms at young and old stages, specifically L3 (larval stage 3) and day 10 (D10) of adulthood. Interestingly, the levels of glycine in *daf-2(e1370)* and *eat-2(ad465)* at D10 were significantly increased relative to both their levels at L3 (S1 Fig). These results suggest that there might be a generic regulatory mechanism mediating the level of glycine during aging in both wild type and long-lived *C*. *elegans*.

To confirm the metabolic branch points of glycine metabolism in the control of glycine levels in *C*. *elegans*, we subjected worms to RNAi against the genes in glycine metabolism (Fig 1A) at the time of hatching, and then measured the level of glycine in D1 worms. Worms treated with RNAi against the genes in glycine synthesis pathways showed no effect on the levels of glycine (Fig 1D). This is perhaps because glycine from bacteria may compensate for the reduction of glycine synthesis in worms. Interestingly, a notable exception to this pattern was found for the knockdown of *mel-32*, encoding a worm homologue of mammalian SHMT1 and SHMT2, which acts to interconvert serine and glycine in one-carbon pathway (Fig 1A) [25]. *mel-32* RNAi led to a strong increase in the level of glycine (Fig 1D) and a concomitant slight decrease in the level of serine (S2 Fig). Thus, these results indicate that MEL-32 is prone to synthesize serine from glycine in *C*. *elegans* (Fig 1D). Likewise, RNAi of *T25B9*.*1* also led to a subtle increase of glycine in worms, suggesting that T25B9.1 tends to degrade glycine in *C*. *elegans* (Fig 1D). RNAi knockdown of genes in glycine catabolic pathways including *daao-1*, *gss-1*, *gcsh-1*, glycine cleavage system T-protein (*gcst-1*), and dihydrolipoamide dehydrogenase (*dld-1*), all significantly increased endogenous glycine compared to control (Fig 1E). Surprisingly, RNAi of phosphoribosylformylglycinamidine synthase (*pfas-1*), which is thought to block glycine being used in purine synthesis, was found to lower the level of glycine (Fig 1E). This implies that there are complex metabolic consequences from the perturbation of *de novo* purine synthesis. Collectively, these data suggest that the majority of glycine in *C*. *elegans* is influenced by two metabolic branches, namely one-carbon metabolism via glycine cleavage complex and serine synthesis via MEL-32.

### Dietary glycine decelerates aging in *C*. *elegans*

We next verified the effects of glycine on lifespan by administering various concentrations of glycine to worms. To avoid influences of glycine on bacterial metabolism and vice versa, we killed *E*. *coli* OP50 with a combination of ultraviolet (UV)-irradiation and antibiotic (carbenicillin) supplementation.

**Fig 2:**
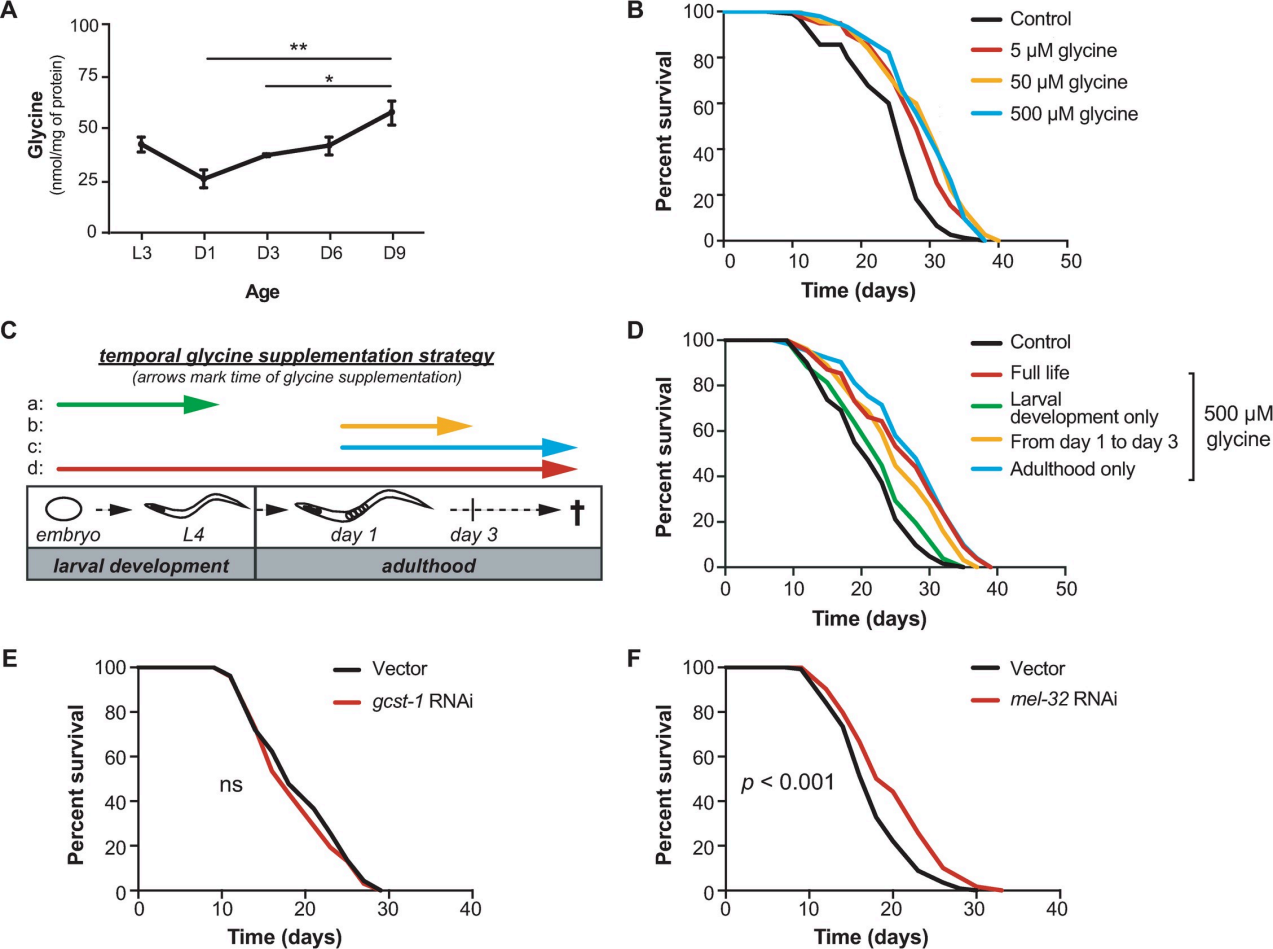
Glycine extends the lifespan of *C*. *elegans*. **(A)** HPLC-MS/MS quantification of glycine in the life history of nematodes fed UV-killed *E*. *coli* OP50 from the larval stage 3 (L3) to D9 adult worms. Levels of other amino acids measured from the same batch of samples were indicated in S4A Fig. Mean ± SD with three biological replicates; Significance was calculated using one-way ANOVA; *p < 0.05; **p < 0.01. **(B)** Lifespan analyses of *C*. *elegans* cultured on UV-killed *E*. *coli* OP50 showing that glycine supplementation significantly extends lifespan at molarities of 5 μM, *p* < 0.0001, 50 μM, *p* < 0.0001, and 500 μM, *p* < 0.0001. Comparisons of survival curves were performed by log-rank tests. Worms were exposed to dietary glycine throughout larval development and the entire adult lifespan. See also S1 Table for lifespan statistics. **(C)** Scheme showing the different timing conditions of glycine treatments. **(D)** Lifespan analyses of *C*. *elegans* fed UV-killed *E*. *coli* OP50 and supplemented with dietary glycine at different times in the animal’s life. Glycine supplementation during larval stage only (from the time of hatching to L4 larval stage) has no effect on lifespan (green line, *p* = 0.18); Glycine supplementation during the first three days of adulthood extends lifespan by 19.4% (yellow line, *p* < 0.001); Glycine supplementation during the adulthood only (from adult D1 until death) extends lifespan by 33.3% (blue line, *p* < 0.0001); Glycine supplementation throughout the entire lifespan (from the time of hatching until death) extends lifespan by 33.3% (red line, *p* < 0.0001). Comparisons of survival curves were performed by log-rank tests. See S1 Table for lifespan statistics. **(E)** Survival curves for worms subjected to *gcst-1* RNAi. Worms were fed HT115 RNAi bacteria expressing dsRNA corresponding to *gcst-1* or empty vector controls from the time of hatching. RNAi depletion of *gcst-1* does not influence the lifespan of the wild-type N2 *C*. *elegans* relative to empty vector controls (*p* = 0.37). Comparisons of survival curves was performed by log-rank tests. See S1 Table for lifespan statistics. **(F)** Survival curves for worms subjected to *mel-32* RNAi. Worms were fed HT115 RNAi bacteria expressing dsRNA corresponding to *mel-32* or empty vector controls from the time of hatching. Knockdown of *mel-32* results in a significant lifespan extension compared with empty vector controls (*p* < 0.001). Comparisons of survival curves was performed by log-rank tests. See S1 Table for lifespan statistics.

In line with a previously reported observation [26], worms being fed UV- and carbenicillin-killed *E*. *coli* OP50 (referred to hereafter as “killed bacteria”) live significantly longer compared to those being fed live *E*. *coli* OP50 (S3 Fig). Therefore, to confirm if glycine still accumulates in aged worms after switching to a killed bacteria diet, we again quantified amino acids levels in worms at different stages of *C*. *elegans* lifespan including L3 (larval stage 3), day 1 (D1), day 3 (D3), day 6 (D6) and day 9 (D9) of adulthood. We found that most amino acids remained unchanged from L3 to D9, including valine, tryptophan, lysine, isoleucine, glutamine, asparagine, aspartate, arginine, serine, and proline (S4A Fig). Some amino acids change with age such as leucine, methionine, ornithine, and glutamate (S4A Fig). The levels of these either peak at the L3 stage or at D3, then decrease with age (S4A Fig). Interestingly, the level of tyrosine peaked at both L3 and D9, and the level of alanine peaked at D3, then maintained stable from D3 to D9 (S4A Fig). Although the levels of some of these amino acids in worms fed killed bacteria were more stable with age compared to those in worms fed live OP50 [22], we consistently found that the levels of leucine, methionine, ornithine, and glutamate decreased and the level of glycine increased in aged worms (Fig 2A and S4A Fig). The results suggest that the changes of these amino acids during aging are robust phenotypes that are independent of the worms being fed live or killed bacteria.

On killed bacteria we tested how a range of glycine concentrations from 5 μM to 10 mM affects the lifespan of *C*. *elegans*. We observed a significant increase in median lifespan at concentrations of 5 μM, 50 μM, and 500 μM of dietary glycine as compared to untreated controls, with a 7.7% (*p* < 0.0001), 19.2% (*p* < 0.0001), and 19.2% (*p* < 0.0001) extension observed, respectively (Fig 2B). Higher concentrations, however, including 5 mM and 10 mM, failed to extend worm lifespan suggesting a dose response where only low concentrations of glycine between 5–500 μM are beneficial to lifespan (Fig 2B, S4B Fig). Our findings are in agreement with previous studies suggesting a dose-effect of amino acids on worm lifespan [12].

Next, we measured amino acid levels in *C*. *elegans* at D1 of the adult stage to investigate how glycine supplementations at different doses affect glycine levels *in vivo*. We did not detect obvious changes of glycine abundance itself or of the other amino acids at any concentration of supplementations such as serine and threonine, the substrates for glycine synthesis (S5A–S5E Fig). These results suggest that in worms, glycine immediately fuels metabolic pathways, likely through glycine cleavage complex and MEL-32 (Fig 1D and 1E). Moreover, the results suggest that the levels of amino acids are tightly controlled in the early adult stage when glycine metabolic genes are actively expressed (Fig 1B and 1C).

To test whether a specific timing is required for the beneficial effect of glycine on *C*. *elegans* lifespan, we administered glycine to worms at different times in the animal’s life. These times included (a) the development phase from the time of hatching to L4, (b) the early adult period from D1 to D3, (c) the adulthood starting from D1 until death, and (d) during the entire life (Fig 2C). Intriguingly, the longevity-promotion function of glycine was observed to act exclusively during adulthood, especially the early adult phase, as supplementation from D1 to D3 was sufficient to prolong lifespan by 19.0% (*p* < 0.0001) (Fig 2D). In contrast, glycine supplementation during development did not exert a beneficial effect on lifespan (Fig 2D). These data suggest that the first three days of adulthood are important for glycine to confer its longevity effects in *C*. *elegans*.

RNAi knockdown of *mel-32* or the components in glycine cleavage complex (e.g. *gcst-1*) resulted in pronounced increases of the endogenous levels of glycine in *C*. *elegans* (Fig 1D and 1E). As such, we wanted to determine whether such increase by blocking two distinct metabolic branches also impacts lifespan in *C*. *elegans*. We hence assayed the lifespan of animals fed RNAi targeting *mel-32* and *gcst-1* from the time of hatching. Intriguingly, we found that while RNAi of *gcst-1* increased the endogenous level of glycine 6.9-fold (Fig 1E), it failed to increase lifespan in *C*. *elegans* (Fig 2E). In contrast, *mel-32* RNAi not only increased the endogenous level of glycine robustly (6.7-fold) (Fig 1D), but also improved the survival of wild-type N2 *C*. *elegans* significantly (Fig 2F). Thus, these results suggest that elevated endogenous or exogenous glycine levels drive lifespan extension, and that an intact glycine cleavage complex is likely required for this extension.

### Glycine supplementation counteracts aging-induced repression of one-carbon and purine metabolism genes

**Fig 3:**
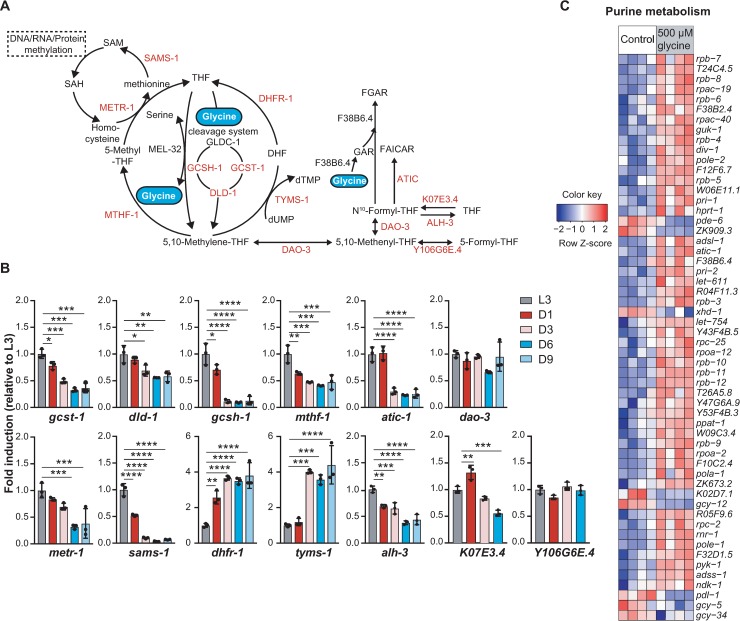
Glycine supplementation counteracts the age-induced suppression of gene expression in purine and one-carbon metabolism. **(A)** Metabolic network of glycine metabolism feeding into one-carbon metabolism and *de novo* purine synthesis. Genes quantified by RT-qPCR and presented in the heat maps are highlighted in red. **(B)** The relative expression levels of the glycine metabolizing genes in worms from larval stage 3 (L3) to D9 adult. Worms were cultured on UV-killed *E*. *coli* OP50 and collected at the desired age. The expression level of the indicated gene was measured by RT-qPCR on total mRNA isolated from synchronized worms at every time point and compared to the mean in L3 worms. Mean ± SD. with three biological replicates; Significance was calculated using one-way ANOVA; *p < 0.5; **p < 0.01; ***p < 0.001; ****p < 0.0001; ns, not significant. **(C)** Heat map showing transcriptional profile of the differentially expressed genes in purine metabolism upon supplementation with 500 μM glycine (z-score normalized). Worms were cultured on UV-killed *E*. *coli* OP50 upon 500 μM glycine treatment from the time of hatching and then collected at adult D1 for total RNA extraction and RNA-sequencing analysis. An adjusted *p*-value < 0.05 was set as the cut-off for significantly differential expression.

As glycine is an important one-carbon donor and an essential substrate for *de novo* purine synthesis, we next sought to understand how one-carbon and purine metabolism are influenced by aging. We therefore quantified the expression levels of genes involved in one-carbon metabolism (Fig 3A) at five developmental distinct time points (L3, D1, D3, D6, and D9) in the worm lifespan. We found that with the exception of *tyms-1* and *dhfr-1* (thymidylate synthetase and dihydrofolate reductase) which were upregulated in aged worms, the expression of genes participating in transferring the one-carbon moiety of glycine to form SAMe including *gcst-1* (glycine cleavage system T-protein), *dld-1* (dihydrolipoamide dehydrogenase), *gcsh-1* (glycine cleavage system H-protein), *mthf-1* (methylene tetrahydrofolate reductase), *metr-1* (methionine synthase), *and sams-1* (S-adenosyl methionine synthetase), dropped dramatically in aged animals (Fig 3B) [27]. Furthermore, the *de novo* purine synthesis genes *atic-1* (5-aminoimidazole-4-carboxamide ribonucleotide formyltransferase/IMP cyclohydrolase) (Fig 3B) and *F38B6*.*4* (Fig 1C) were markedly reduced in aged worms compared to their expression levels in worms at D1. Together these data suggest a downregulation of both one-carbon and purine metabolism during aging in *C*. *elegans*.

To resolve in greater detail how glycine supplementation counteracts the age-related changes in one-carbon and purine metabolic genes, we performed next generation RNA-sequencing on D1 worms which were fed control diet (UV-killed *E*. *coli* OP50) and 500 μM glycine-supplemented diet from the time of hatching, respectively. In contrast to the changes occurring with age, glycine induced a marked increase in the expression of genes in purine metabolic pathways (Fig 3C). Concomitantly, several genes in one-carbon metabolism (indicated in red in S6 Fig) were differentially expressed, including upregulation of *atic-1*, *F38B6*.*4*, *dhfr-1*, *tyms-1*, *mel-32*, and *gcsh-1*, and downregulation of *sams-1*, *mthf-1*, and *gldc-1* (S6 Fig). These results suggest that the expression of purine metabolism genes is subjected to an overall upregulation by exogenous supplementation of dietary glycine, while genes in one-carbon metabolism are under complex regulations upon glycine supplementation.

### Transcriptional upregulation of glycine and one-carbon metabolism is a shared signature of longevity

Having determined one of the effects of glycine on worm lifespan and its ability to partly counteract age-related declines in gene expressions in one-carbon and purine metabolic pathways, we next aimed to investigate whether similar gene regulatory events are also present in long-lived mutant worms. To test this, we turned to microarray datasets from three long-lived worm models, *daf-2(e1370)* and *eat-2(ad465)* [28] or *mrps-5* RNAi worms (reported here). We specifically looked at glycine-associated metabolic pathways in the Kyoto Encyclopedia of Genes and Genomes (KEGG) database including ‘glycine serine and threonine metabolism’, ‘one carbon pool by folate’, ‘cysteine and methionine metabolism’, and ‘purine metabolism’. Strikingly, although distinct longevity pathways are known to be active in these long-lived worms [2,29,30], all these longevity worm models consistently showed a transcriptional activation of glycine metabolism, folate-dependent one-carbon metabolism, and methionine metabolism (S7A–S7C Fig). This suggests that transcriptional activation of glycine and one-carbon metabolism is a prominent signature of longevity shared by these long-lived worms. In contrast, overall purine metabolism including *de novo* synthesis, the salvage pathways, and purine degradation was only mildly deactivated across the three long-lived strains (S7A–S7C Fig).

To identify prominent transcriptional features in the three long-lived strains, we next examined the expression profile of individual genes belonging to “glycine, serine and threonine metabolism”, as well as folate-mediated one-carbon metabolism and purine metabolism. We found that genes involved in glycine anabolism including *T09B4*.*8*, *agxt-1*, *C15B12*.*1*, *R102*.*4*, and *T25B9*.*1* were upregulated in *daf-2(e1370)*, eat-2*(ad465)* [28] and *mrps-5* RNAi worms (S7D–S7F Fig). Moreover, a concomitant rise in the expression levels of genes in glycine catabolism and *de novo* purine synthesis occurred, including *gldc-1*, *gcst-1*, *gcsh-1*, *F38B6*.*4*, and *atic-1* (S7D–S7I Fig), suggesting a stimulation of metabolic activity of glycine-associated processes in these long-lived worms. To query the expression of genes in the production of SAMe in the long-lived worm models, we specifically checked the expression of five homologues of SAMe synthetases in *C*. *elegans* from the microarray data including *sams-1*, *sams-2*, *sams-3*, *sams-4*, and *sams-5* (S8 Fig). Interestingly, the expression of *sams-1*, which encodes the enzyme accounting for the majority of overall SAMe production in *C*. *elegans* [31], was significantly upregulated in all these long-lived worms (S8 Fig). Moreover, *sams-5* was increased in *daf-2(e1370)* and *eat-2(ad465)* [28], while *sams-2*, *sams-3*, and *sams-4* were suppressed in *mrps-5* RNAi and *daf-2(e1370)* (S8 Fig). Collectively, the data point to elevated methylation activities in these long-lived worms.

In contrast to an overall upregulation of purine metabolism genes in response to dietary glycine treatment, all three longevity worm models specifically induced the expression of genes in *de novo* purine synthesis compared to control, including *F38B6*.*4*, *F10F2*.*2 (pfas-1*, phosphoribosylformylglycinamidine synthase*)*, *B0286*.*3* (*pacs-1*, phosphoribosylaminoimidazole succinocarboxamide synthetase), and *atic-1* (S9A–S9C Fig).

Taken together, our data reveal that transcriptional activations of glycine and glycine-associated pathways, including one-carbon and *de novo* purine synthesis, are present in three distinct longevity models.

### Dietary glycine causes large-scale changes in gene expression

**Fig 4:**
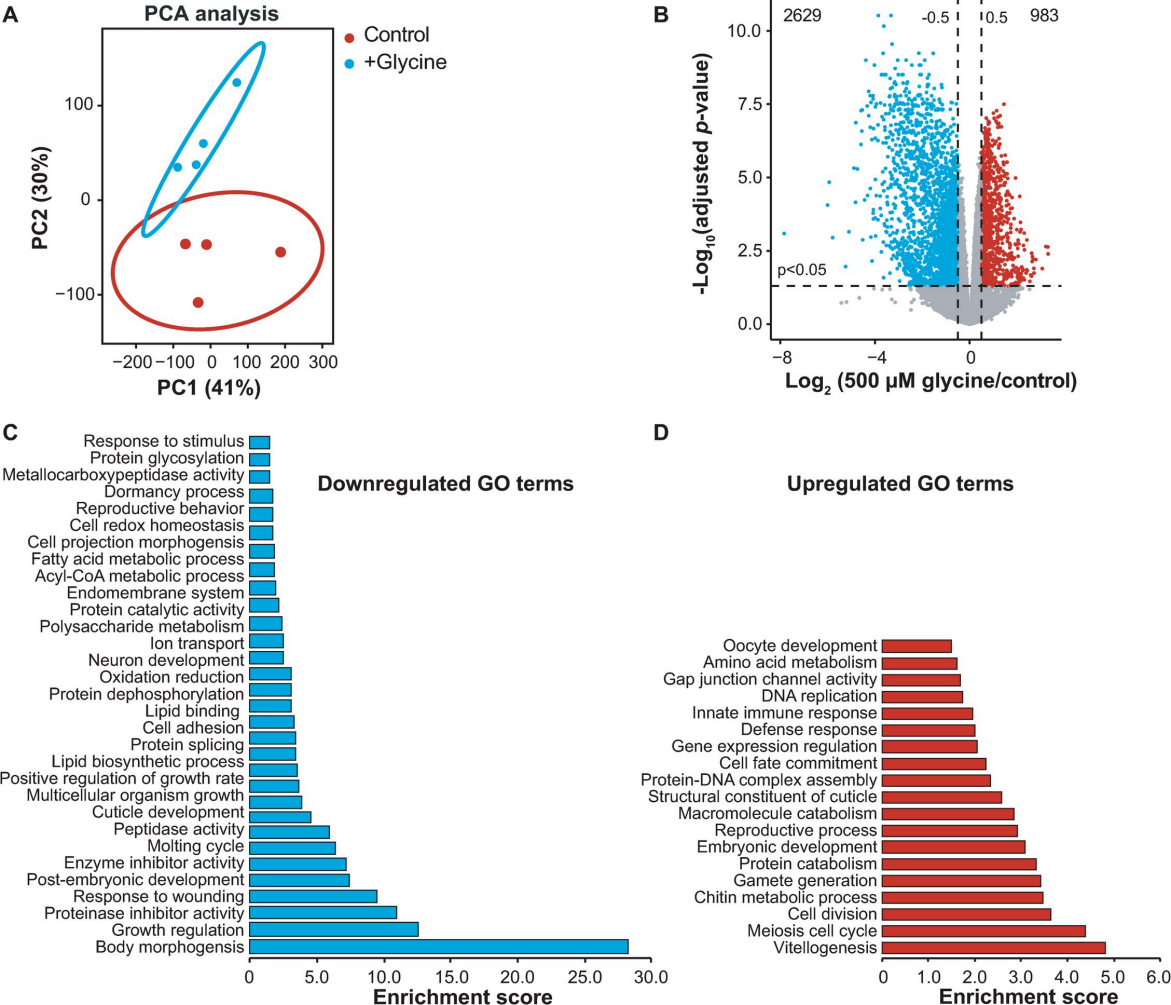
Genome-wide analyses identify cellular metabolic processes affected by glycine supplementation. **(A)** Principle Component Analysis (PCA) showing group separation based on differentially expressed genes in 500 μM glycine supplemented worms compared to controls. Worms were cultured on UV-killed *E*. *coli* OP50 upon 500 μM glycine treatment from the time of hatching and then collected at adult D1 for total RNA extraction and RNA-sequencing analysis with four biological replicates per condition. **(B)** Differentially expressed genes in 500 μM glycine compared to controls visualized with a volcano plot. An adjusted *p*-value < 0.05 and |log_2_ (500 μM glycine / control ratio) | > 0.5 were applied to determine significantly up- and downregulated genes (red and blue dots), or genes without change in expression (grey dots). **(C and D)** Functional annotation clustering for the 2629 significantly downregulated genes **(C)** and the 973 significantly upregulated genes (blue and red dots from the volcano plots B with a *p*-value < 0.05) **(D)** determined using DAVID Bioinformatics Database with an EASE score < 0.05.

To understand the mechanism of glycine-mediated lifespan extension on a more global scale, we returned to our next-generation RNA-sequencing dataset and performed unsupervised Principle Component Analysis (PCA) on the individual libraries. We found a clear separation between glycine-treated versus untreated samples (Fig 4A), corresponding to a large difference in gene expression (Fig 4B). Interestingly, more genes were transcriptionally repressed in response to glycine treatment in which 2629 genes were differentially down-regulated, and 983 genes were up-regulated compared to control (Fig 4B). This suggests an inhibition propensity of glycine on gene expression.

To probe the processes changed upon glycine supplementation, we performed gene ontology (GO) term enrichment analysis on the differentially expressed genes using the Database for Annotation, Visualization and Integrated Discovery (DAVID) bioinformatics resource [32]. A larger number of GO terms were found enriched in the downregulated gene list, among which were GO terms for body morphogenesis, growth regulation, post-embryonic development, molting cycle, cuticle development, multicellular organism growth, and positive regulation of growth rate (Fig 4C). These enrichments are all related to growth control, suggesting a decelerating effect of glycine upon development and growth which is a phenomenon known to be associated to longevity [33]. Additionally, unlike some mutations that confer longevity to the soma at the cost of a reduction in fecundity [34], supplementation of glycine mildly enhanced the expression of genes in reproduction-related biological processes such as the GO terms of vitellogenesis, meiosis cell cycle, and gamete generation (Fig 4D). While we did not observe clear differences in the number or the size of embryos, the progenies from glycine- and serine-supplemented worms seem to be healthy and normal. Overall, with the observations of the lifespan extending effects of glycine, these data suggest that the beneficial role of glycine slows down pathways that are traditionally ameliorated in healthy aging models.

### Methionine cycle integrity is required for glycine-mediated lifespan extension

**Fig 5:**
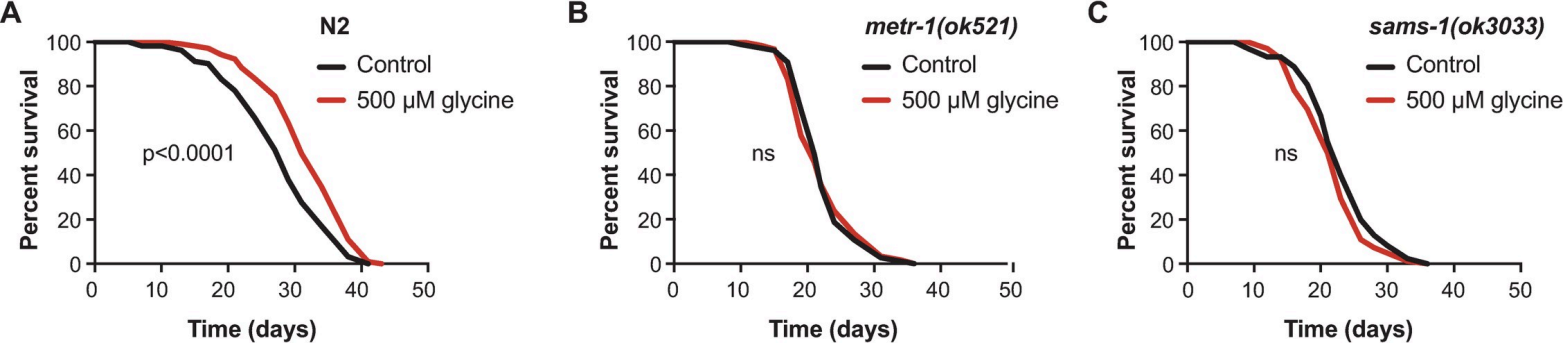
The methionine cycle is required for the longevity effect of glycine in *C*. *elegans*. **(A)** The lifespan analysis of N2 worms fed control diet (UV-killed *E*. *coli* OP50) or 500 μM glycine diet showing that 500 μM glycine supplementation significantly promotes lifespan (*p* < 0.0001). **(B and C)** Lifespan analysis of the mutant *metr-1(ok521)* worms or **(B)** *sams-1(ok3033)* worms **(C)** supplemented with 500 μM glycine diet compared to their corresponding mutant worms fed control diet (UV-killed *E*. *coli* OP50). Comparisons of survival curves were performed by log-rank tests. See S1 Table for lifespan statistics.

MEL-32 and glycine cleavage complex are major gatekeepers responsible for the flux of glycine into metabolic pathways (Fig 1A). Particularly, although suppression of *mel-32* or the components in glycine cleavage complex in *C*. *elegans* dramatically elevated the level of endogenous glycine (Fig 1D and 1E), only *mel-32* RNAi showed a potent lifespan extension effect on worms likely by favoring the flux of glycine into one-carbon metabolism (Fig 2E and 2F). Additionally, by fueling the one-carbon metabolic network through glycine cleavage complex, glycine contributes to the synthesis of SAMe (Fig 3A), the availability of which has been implicated in the regulation of histone methylation patterns and subsequently gene expressions [35]. Collectively, this led us to hypothesize that the methionine cycle may be required for the lifespan-extending effect of glycine. To test if the methionine cycle is necessary for the longevity effect of glycine, we performed lifespan analyses with the methionine cycle-deficient mutants *metr-1(ok521)* and *sams-1(ok3033)*. In these mutants, 500 μM glycine failed to promote lifespan (Fig 5A–5C), demonstrating that the effects of glycine on worm longevity depend on the methionine cycle.

### Serine shares the conserved mechanism with glycine to extend lifespan

**Fig 6:**
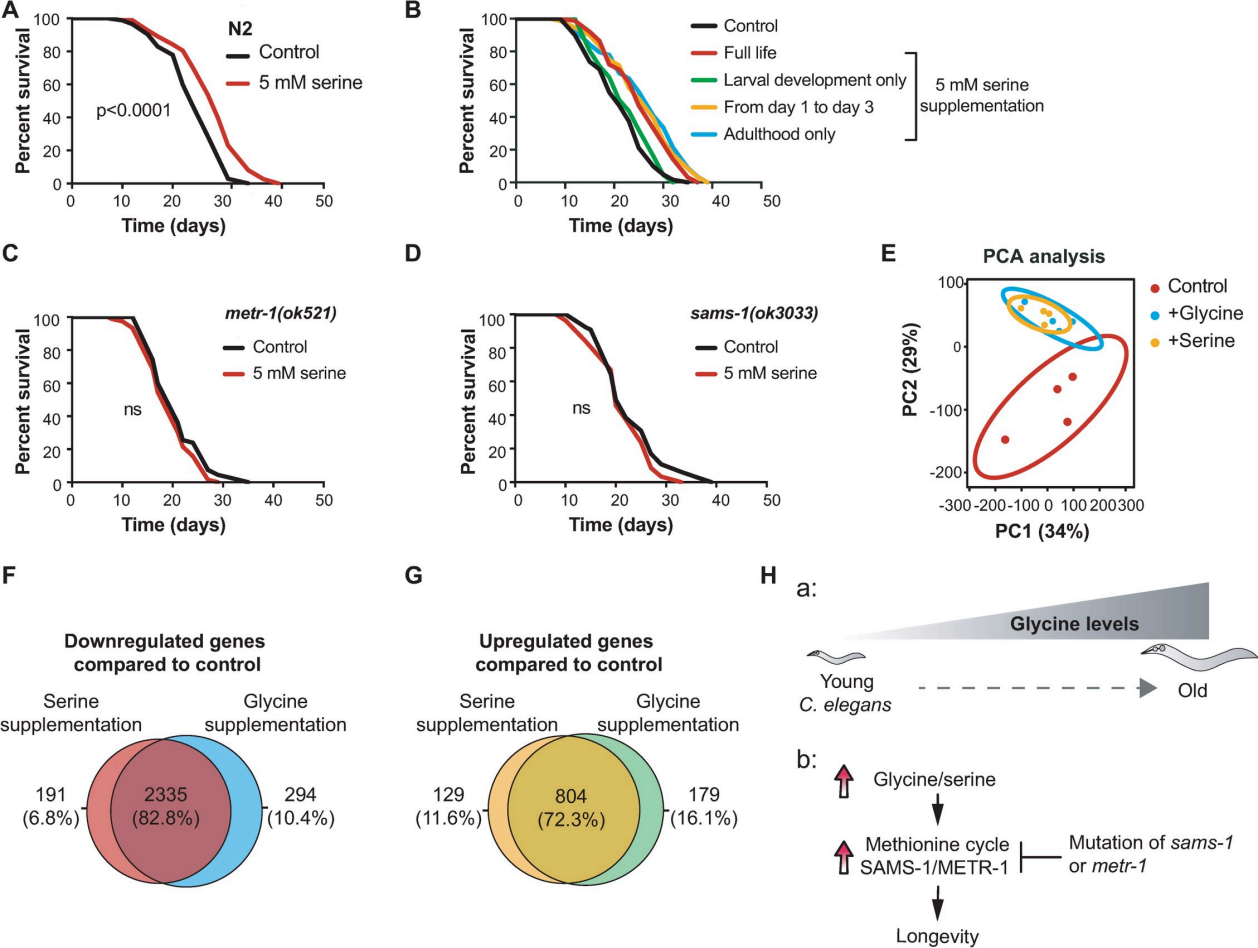
Serine increases *C*. *elegans* lifespan in a methionine cycle-dependent fashion. **(A)** Lifespan analysis of N2 showing that 5 mM serine promotes longevity (*p* < 0.0001). Worms were cultured on UV-killed *E*. *coli* OP50 upon 5 mM serine treatment from the time of hatching. **(B)** Lifespan analyses performed in N2 worms fed UV-killed *E*. *coli* OP50 and supplemented with 5 mM serine at different time points. Serine supplementation during the larval stage only (from egg until L4 larval stage) has no effect on lifespan (green line, *p* = 0.32). Serine supplementation during the early adult phase from D1 to D3 extends lifespan by 33.3% (*p* < 0.0001, yellow line); Serine supplementation during the adulthood only (from adult D1 until death) extends lifespan by 33.3% (*p* < 0.0001, blue line). Serine supplementation throughout the entire life (from eggs until death) extends lifespan by 19.0% (*p* < 0.0001, red line). **(C and D)** Lifespan analysis performed in *metr-1(ok521)* **(C)** and *sams-1(ok3033)* mutant worms **(D)** supplemented with 5 mM serine compared to their corresponding mutant worms fed control diet (UV-killed *E*. *coli* OP50). Comparisons of survival curves were performed by log-rank tests. See S1 Table for lifespan statistics. **(E)** Principle Component Analysis (PCA) plot showing group separation based on transcriptional profiles in worms supplemented with 500 μM glycine or 5 mM serine compared to control worms fed UV-killed *E*. *coli* OP50. Worms were supplemented with 500 μM glycine or 5 mM serine from the time of hatching and collected at adult D1 for total RNA extraction and RNA-sequencing analysis with four biological replicates per condition. **(F and G)** Venn diagrams comparing overlaps between genes significantly downregulated or upregulated in glycine and serine supplementation groups compared to control worms fed UV-killed *E*. *coli* OP50. 2335 downregulated genes **(F)** and 804 upregulated genes **(G)** are found to be shared by glycine and serine supplementation groups. An adjusted *p*-value < 0.05, |log_2_ (500 μM glycine / control ratio)| > 0.5, |log_2_ (5 mM serine / control ratio)| > 0.5 were used to determine differentially expressed genes. **(H)** Model for how glycine and serine supplementation modulate longevity in *C*. *elegans*. a. Illustration depicting the relationship between glycine levels and aging in *C*. *elegans*; b. Model illustrating how glycine supplementation may function through methionine cycle to increase lifespan and how mutation of *metr-1* or *sams-1* affects outcome.

Serine is another important one-carbon donor and the major precursor for glycine synthesis *in vivo* [14]. Thus, serine and glycine are closely related to each other in one-carbon metabolism. Given their similarities, we next investigated whether serine can also exert beneficial effects on worm lifespan. Serine supplementation at a concentration from 1 mM to 10 mM has been shown previously to extend worm lifespan [12]. We therefore administered 5 mM serine to worms and measured lifespan. Similar to glycine, we confirmed that serine prolonged the lifespan of worms (+20.8% in median lifespan) (Fig 6A).

Given that the adulthood is important for glycine-mediated lifespan extension, we investigated whether serine increases lifespan in the same fashion as glycine. Similarly, we treated worms with serine at different times in worm’s lifespan including (a) developmental stage, (b) the beginning of adulthood from D1 to D3, (c) adulthood from D1 until death, and (d) during the whole lifetime. Similar to the effects of glycine on lifespan, serine treatment from D1 to D3 is sufficient to recapitulate the beneficial effects on lifespan as did the treatment throughout the entire life or during adulthood only (Fig 6B). In contrast, supplementation during the developmental stage failed to increase lifespan (Fig 6B). This further implies that serine acts on the same downstream longevity signalling pathways to influence aging as does glycine.

We further investigated whether the anti-aging effects of serine also rely on the methionine cycle. In agreement with the results observed with glycine supplementation, the lifespan extending effect of serine was also ablated by mutations of *metr-1* and *sams-1* (Fig 6C and 6D). These results further suggest that serine and glycine prolong *C*. *elegans* lifespan via a similar mechanism.

To determine the common regulators in both glycine and serine-mediated longevity, we performed RNA-sequencing on serine-supplemented worms. As expected, PCA analysis showed a clear separation between worms treated with either of the amino acids when compared to non-treated worms, and a strong similarity between glycine- and serine-treated worm groups (Fig 6E). Statistical analysis found one significantly differentially expressed gene, *F38B6*.*4* (S10A Fig), an enzyme that consumes glycine for purine synthesis. In addition, visualizing the data as a volcano plot showed again a greater number of genes repressed by serine treatment (2865 downregulated genes vs 973 upregulated genes), in line with the same gene expression suppression pattern of glycine-supplemented worms (S10B Fig, Fig 4B). Likewise, we found a strong overlap between glycine- and serine-treated worms when looking at the up- and downregulated genes, as shown in the Venn diagrams where 82.8% (2335) of the downregulated and 72.3% (804) of the upregulated genes are shared (Fig 6E–6G). Taken together, we suggest a model whereby both glycine and serine supplementation stimulate longevity in a methionine cycle-dependent fashion and through common signaling pathways. Moreover, this seems to be dependent on the expression of *sams-1* and *metr-1*. This model is illustrated in Fig 6H.

## Discussion

Our work sheds light onto the means by which the amino acid glycine can increase *C*. *elegans* lifespan when supplemented to the diet. Using a metabolomics approach, we found that glycine steadily and significantly accumulates in aging *C*. *elegans* [22]. Furthermore, we demonstrated that this accumulation is mainly coupled to a decrease in the expression levels of genes in glycine cleavage pathway which control the majority of glycine breakdown in *C*. *elegans*. We found that *mel-32* RNAi causes a marked rise in the endogenous level of glycine which in turn extends lifespan. Moreover, supplementing dietary glycine extends lifespan at concentrations between 5–500 μM, while mutations in methionine synthase [*metr-1(ok521)*] and S-adenosyl methionine synthetase [*sams-1(ok3033)*], two enzymes involved in methionine cycle, can fully abrogate this lifespan extension. Furthermore, we found that serine, another amino acid that feeds into one-carbon metabolism, shows similar transcriptional changes, *metr-1* and *sams-1* dependency, and lifespan extension upon dietary supplementation as does glycine. These results confirm an important role for the methionine cycle in the longevity effects of glycine.

Our work reveals a timing requirement of glycine supplementation in the promotion of longevity in *C*. *elegans*. Specifically, the first three days of adulthood (from D1 to D3) are crucial for glycine to confer the benefits on longevity. Given that the DAF-2 pathway also acts exclusively during adulthood and throughout the reproductive period to affect lifespan in *C*. *elegans* [36], further investigation is warranted to see if this classical longevity pathway is fully or only partially required for the beneficial effects of glycine. Furthermore, the first three days of adulthood coincide with the reproductive period of worms, raising an interesting question for future studies about the crosstalk between the reproductive system and glycine-activated longevity pathways.

Our work identified a counterintuitive biological phenomenon, whereby glycine accumulation was observed during the aging process in worms while supplementation of glycine was nonetheless able to prolong worm lifespan. However, it is not uncommon for changes that occur with age to also benefit lifespan when artificially induced. For example, suppression of IGF1 signaling may extend lifespan in many model organisms [2,37,38], while IGF1 levels themselves have been observed to decline with age [39,40]. Moreover, methionine restriction is beneficial to lifespan in a variety of model organisms [11,41], while methionine abundance *in vivo* has been observed to decline with age [observed in this study (S4A Fig) and [22]]. Similar to these phenomena, glycine supplementation may activate protective cellular pathways that promote longevity when exogenously applied, while a natural glycine accumulation with age may reflect the organism’s need to upregulate these same cytoprotective pathways to deal with the damage and detrimental changes occurring during aging.

Studies in rodents have suggested glycine supplementation to have pro-longevity effects [42,43], anti-inflammatory effects [44], to be cytoprotective [45], and to ameliorate metabolic disorders [46]. In humans, glycine supplementation in patients with metabolic disorders has a protective effect against oxidative stress and inflammation [47–49]. In line with these observations in mammalian systems, our data demonstrated that glycine promotes longevity in *C*. *elegans*. Furthermore, we show this benefit to occur in a *metr-1* and *sams-1* dependent manner, implicating the methionine cycle in longevity regulation. Finally, we show glycine supplementation induces widespread suppression of genes including many that are hallmarks of the aging process. Taken together, our findings suggest that dietary glycine is an effective strategy to increase lifespan and warrant further investigation for life- and healthspan studies in humans.

## Materials and methods

### Worm strains and maintenance

*C*. *elegans* strains N2 Bristol, RB2204 *sams-1(ok3033)X*, RB755 *metr-1(ok521)II*, *eat-2(ad465)*, and *daf-2(e1370)* were obtained from the Caenorhabditis Genetics Center (CGC, University of Minnesota). Nematodes were grown and maintained on Nematode growth media (NGM) agar plates at 20°C as previously described [50].

### Bacterial feeding strains and RNAi experiments

*E*. *coli* OP50 and *E*. *coli* HT115 (DE3) with the empty vector L4440 was obtained from the CGC. Bacterial feeding RNAi experiments were carried out as described [51]. RNAi *E*. *coli* feeding clones used were *mrps-5* (E02A10.1), *T09B4*.*8*, *agxt-1* (T14D7.1), *C15B12*.*1*, *R102*.*4*, *T25B9*.*1*, *gss-1* (M176.2), *gcsh-1* (D1025.2), *gcst-1* (F25B4.1), *pfas-1* (F10F2.2), and *dld-1* (LLC1.3). Clones of *agxt-1* (T14D7.1), *C15B12*.*1*, *R102*.*4*, *T25B9*.*1*, *gss-1* (M176.2), *gcsh-1* (D1025.2), *gcst-1* (F25B4.1), and *pfas-1* (F10F2.2) were derived from the Ahringer RNAi library [52]; Clones of *mrps-5* (E02A10.1), *T09B4*.*8*, and *dld-1* (LLC1.3) were derived from the Vidal RNAi library [53]; Worms were fed RNAi bacteria from the time of hatching unless otherwise indicated.

### Preparation of amino acid supplementation plates

Glycine and serine were purchased from Merck Millipore (no. 8.1603.0250) and Sigma (no. S4500), respectively. A stock of concentration of 1 M glycine and serine was made by dissolving glycine and serine in water. The pH of glycine and serine stock solution was adjusted to 6.0–6.5 with sodium hydroxide and then sterilized with 0.45 μm Millipore filter. The concentrations of 5 μM, 50 μM, 500 μM, 5 mM and 10 mM glycine, and 5 mM serine were used in the present study.

### Preparation of UV-killed bacteria

Overnight cultures of *E*. *coli* OP50 were seeded on standard NGM plates containing carbenicillin (25 μg ml^-1^) to prevent the bacterial growth. After drying overnight at room temperature, the bacterial lawn was irradiated with 254 nm UV light using a Stratalinker UV Crosslinker model 1800 (Stratagene, USA) at 999900 μJ/cm^2^ for 5 min. A sample of UV-exposed *E*. *coli* OP50 was collected and cultured in LB medium overnight at 37°C to confirm the bacteria were completely killed. Plates seeded with UV-killed bacteria were stored in 4°C and used within 1 week after seeding.

### Lifespan measurements

Lifespan experiments of amino acids supplementation were performed at 20°C without fluorouracil as described with the exception that UV-killed *E*. *coli* OP50 was used [54]. Briefly, for treatment throughout lifespan, worms were cultured on glycine- or serine- supplemented plates from the time of hatching until death; For the treatment during larval development only, worms were cultured on glycine- or serine- supplemented plates from egg until L4, and then transferred onto control plates until death; For the treatment from D1 to D3, worms were cultured on glycine- or serine- supplemented plates from D1 (one day after L4) to D3, and transferred on control plates. For amino acid treatment during adulthood only, worms were cultured on glycine or serine supplemented plates from D1 until death. During the reproductive period (≈ day 1–8), worms were transferred to fresh plates every other day to separate them from their progeny.

For RNAi lifespan experiments, worms were cultured on NGM plates containing 2mM IPTG and seeded with HT115 (DE3) bacteria transformed with either pL4440 empty vector or the indicated RNAi construct from the time of hatching. Worms were transferred to fresh plates containing 10 μM fluorouracil at L4 larval stage to prevent egg laying. 100–150 worms per condition were used for every lifespan. Survival was scored every other day throughout the lifespan and a worm was considered as dead when they did not respond to three taps. Worms that were missing, displaying internal egg hatching, losing vulva integrity, and burrowing into NGM agar were censored. Statistical analyses of lifespan were calculated by Log-rank (Mantel-Cox) tests on the Kaplan-Meier curves in GraphPad Prism.

### Amino acid extraction and UPLC-MS/MS analysis

Amino acids were extracted and analyzed as described before [22] and each experiment was performed in three biological replicates. About amino acids profiles change with age in wild type N2 related to Fig 2A and S4A Fig, worms were cultured on UV-killed *E*. *coli* OP50 and collected at the desired stage (L3, D1, D3, D6, and D9) for amino acids extraction; About amino acids profiles change with age in wild type N2, *daf-2(e1370)*, and *eat-2(ad465)* related to S1 Fig, worms were cultured on alive *E*. *coli* OP50 and collected at L3 and D10 for amino acids extraction; About glycine supplementation experiments related to S5A–S5E Fig, worms were fed UV-killed *E*. *coli* OP50 and supplemented with glycine at the desired concentration (5 μM, 50 μM, 500 μM, 5 mM, and 10 mM) or with water control from the time of hatching. Then, D1 worms were harvested for amino acids extraction; About RNAi experiments related to Fig 1D and 1E, and S2 Fig, worms were cultured on NGM plates containing 2mM IPTG and seeded with HT115 (DE3) bacteria transformed with either pL4440 empty vector or the indicated RNAi construct from the time of hatching. Then, D1 worms were harvested for amino acids extraction.

Around 1500 synchronized worms at the desired stage were collected, freeze-dried and stored at room temperature until use. Worm lysates were obtained by homogenization and subsequent tip sonication. Amino acids were extracted from worm lysate containing 50 μg protein and measure by UPLC-MS/MS analysis.

### Extraction of mRNA and quantitative real-time PCR (qPCR) for gene expression in *C*. *elegans*

In Fig 1B and 1C, worms were cultured on live *E*. *coli* OP50 and harvested at D1, D4, and D9 for mRNA extraction; In Fig 3B, worms were cultured on UV-killed *E*. *coli* OP50 and collected at L3, D1, D3, D6, and D9 for mRNA extraction. Approximately 500 worms were collected in three biological replicates per condition at the desired stage. Total RNA was isolated according to the manufacturer’s protocol. Briefly, samples were homogenized in TRIzol (Invitrogen) with a 5 mm steel metal bead and shaken using a TissueLyser II (Qiagen) for 5 min at a frequency of 30 times/sec. RNA was quantified with a NanoDrop 2000 spectrophotometer (Thermo Scientific) and stored at -80°C until use. Genomic DNA was eliminated, and cDNA was synthesized using the QuantiTect Reverse Transcription kit (QIAGEN). The qPCR reaction was carried out in 8 μL with a primer concentration of 1 μM and SYBR Green Master mix (Roche) in a Roche LightCycler 480 system. In all analyses, the geometric mean of two reference genes, *eif-3*.*C* and F35G12.2, was used for normalization and the oligonucleotides used for PCR are listed in S2 Table.

### Microarray

*daf-2(e1370)* and *eat-2(ad465)* worms were fed HT115 *E*. *coli* expressing empty vector from the time of hatching. Wild type N2 worms were fed HT115 *E*. *coli* expressing dsRNA against *mrps-5* from the larval stage 4 of parental worms, and this exposure was continued in the first filial population (F1). *mrps-5* RNAi-treated F1 worms were used for total RNA extraction. Microarray experiment was performed as described [28]. Approximately 500 young adult worms were collected in four replicates per condition and total RNA was extracted as described above. RNA quality and quantity were assessed after DNase clean-up using a 2100 Bioanalyzer (Agilent Technologies). RNA was amplified and labeled using a Low Input QuickAmp Labeling Kit (Agilent Technologies) and hybridized using the Agilent Gene Expression Hybridization Kit (Agilent Technologies). An ArrayXS-068300 with WormBase WS241 genome build (OakLabs) was used and fluorescence signals were detected by the SureScan microarray Scanner (Agilent Technologies). Data of all samples were quantile normalized using the ranked median quantiles as described previously [55].

### Samples for RNA library preparation

Worms were cultured on UV-killed *E*. *coli* OP50 upon 500 μM glycine treatment or 5 mM serine treatment from the time of hatching. Approximately 500 worms at D1 were collected in quadruplicates per condition for total RNA extraction as described above. Genomic DNA residues were eliminated with RNase-Free DNase (Qiagen), followed with the cleaning up with the RNeasy MinElute Cleanup Kit (Qiagen). Samples were sent to GenomeScan B.V. (Leiden, The Netherlands) for RNA library preparation and sequencing at a 20 million read-depth (see methods below).

### RNA library preparation and next-generation sequencing (RNA-seq)

Samples were processed for Illumina using the NEBNext Ultra Directional RNA Library Prep Kit (NEB #E7420) according to manufacturer’s description. Briefly, rRNA was depleted using the rRNA depletion kit (NEB# E6310). A cDNA synthesis was performed in order to ligate with the sequencing adapters. Quality and yield after sample preparation was measured with the Fragment Analyzer. Size of the resulting products was consistent with the expected size distribution (a broad peak between 300–500 bp). Clustering and DNA sequencing using the Illumina cBot and HiSeq 4000 was performed according to manufacturer's protocol with a concentration of 3.0 nM of DNA. HiSeq control software HCS v3.4.0, image analysis, base calling, and quality check was performed with the Illumina data analysis pipeline RTA v2.7.7 asnd Bcl2fastq v2.17.

### RNA-seq: Read mapping, statistical analyses, and data visualization

Reads were subjected to quality control FastQC (http://www.bioinformatics.babraham.ac.uk/projects/fastqc), trimmed using Trimmomatic v0.32 [56] and aligned to the *C*. *elegans* genome obtained from Ensembl, wbcel235.v91 using HISAT2 v2.0.4 [57]. Counts were obtained using HTSeq (v0.6.1, default parameters) [58] using the corresponding GTF taking into account the directions of the reads. Statistical analyses were performed using the edgeR [59] and limma/voom [60] R packages. All genes with no counts in any of the samples were removed whilst genes with more than 2 reads in at least 4 of the samples were kept. Count data were transformed to log2-counts per million (logCPM), normalized by applying the trimmed mean of M-values method (Robinson et al., 2010) and precision weighted using voom [61]. Differential expression was assessed using an empirical Bayes moderated *t*-test within limma’s linear model framework including the precision weights estimated by voom [61]. Resulting *p-*values were corrected for multiple testing using the Benjamini-Hochberg false discovery rate. Genes were re-annotated using biomaRt using the Ensembl genome databases (v91). RNA-seq samples were compared using principal component analysis (PCA) and Partial least squares discriminant analysis (PLS-DA) using mixomics [62]. Heatmaps, venn diagrams, and volcano plots were generated using ggplot2 [63] in combination with ggrepel (https://CRAN.R-project.org/package=ggrepel), and venneuler (https://CRAN.R-project.org). Data processing and visualization was performed using R v3.4.3 and Bioconductor v3.6.

### Functional annotation

Gene ontology (GO) analyses were conducted using DAVID bioinformatics resource [32]. Genes found to be significantly up- or downregulated with an adjusted *p*-value < 0.05 and | log_2_ (fold change) | > 0.5 were subjected to functional annotation clustering. To retrieve significantly enriched GO terms, enrichment threshold (EASE score) was set as 0.05 for all analyses and the category of each annotation cluster generated by David was curated manually.

### Gene expression profile visualization

Heat maps of gene expression profile in S7–S9 Figs were plotted using “R2”, Genomics analysis and Visualization platform (http://r2.amc.nl). Gene set map analyses were performed on “R2” with defined gene sets from the Kyoto Encyclopedia of Genes and Genomes (KEGG) pathways (http://www.genome.jp/kegg/) [64].

### Quantification and statistical analysis

Data were analyzed by two-tailed unpaired Student’s *t*-test or by one-way ANOVA with Tukey’s post hoc test for multiple comparisons, except for survival curves, which were calculated using the log-rank (Mantel-Cox) method. For all experiments, data are shown as mean ± SD and a *p*-value < 0.05 was considered significant.

## Supporting information

S1 Fig The level of glycine shows an increasing trend with age in *daf-2(e1370)* and *eat-2(ad465)*. The levels of glycine were quantified by UPLC-MS/MS in young (L3) and aged (D10) wild type N2, *daf-2(e1370)* and *eat-2(ad465)* animals. The level of glycine is significantly higher at the age of D10 relative to the level of glycine at L3 in N2, *daf-2(e1370)* and *eat-2(ad465)*. Worms were cultured on live *E*. *coli* OP50 and collected at the desired stage for amino acids extraction. Bar graphs are expressed as mean ± SD with three biological replicates; Significance was calculated using Student’s *t*-test; *p < 0.05, **p < 0.01.
(TIF)

S2 Fig RNAi knockdown of *mel-32* decreased the level of serine in *C. elegans*. The level of serine measured by UPLC-MS/MS in worms subjected to RNAi against *mel-32*. Worms were fed HT115 RNAi bacteria against *mel-32* from the time of hatching and collected for amino acids extraction at D1. Bar graphs are expressed as mean ± SD with four biological replicates; Significance was calculated using Student’s *t*-test; ***p < 0.001.
(TIF)

S3 Fig UV-killed bacteria extends the lifespan of *C. elegans*. Lifespan analysis of *C*. *elegans* cultured on alive and UV-killed *E*. *coli* OP50, showing that the latter lives longer. Comparisons of survival curves was performed by log-rank tests. See S1 Table for lifespan statistics.
(TIF)

S4 Fig Amino acids profiles change with age in *C. elegans*. **(A)** Amino acid amounts were measured using UPLC-MS/MS across 5 time-points of the lifespan of worms fed UV-killed *E*. *coli* OP50 with three biological replicates, including L3, D1, D3, D6, and D9. Note: the profile of glycine is shown in Fig 2A. Statistical analysis between groups was performed using one-way ANOVA. Significance levels are indicated with asterisks as follows: *p < 0.05; **p < 0.01, ***p < 0.001. **(B)** Lifespan analyses of *C*. *elegans* cultured on UV-killed *E*. *coli* OP50 upon 5 mM and 10 mM glycine treatments showing that glycine supplementation at 5 mM and 10 mM has no effect on lifespan. Comparisons of survival curves was performed by log-rank tests. See S1 Table for lifespan statistics.
(TIF)

S5 Fig Exogenous glycine supplementation does not alter amino acid profile in day 1 adult worms. **(A-D)** Amino acid profile of D1 adult worms supplemented with increasing amounts of glycine ranging from 5 μM to 10 mM and fed UV-killed *E*. *coli* OP50. The levels of glycine, serine, methionine and threonine in **(A)** are presented in separate graphs **(B)**, **(C)**, **(D)**, and **(E)** with the levels of significance indicated. Bar graphs are expressed as mean ± SD with three biological replicates; Statistical analysis was performed using one-way ANOVA. Significance levels are indicated with asterisks as follows: ns, not significant; *p < 0.05; **p < 0.01.
(TIF)

S6 Fig Glycine supplementation counteracts the age-induced suppression of gene expression in one-carbon metabolism. Heat map showing transcriptional changes of the genes in the Fig 3A diagram upon supplementation with 500 μM glycine (z-score normalized). Worms were fed UV-killed *E*. *coli* OP50 upon 500 μM glycine treatment from the time of hatching, and collected at adult D1 for total RNA extraction, then continued with RNA-sequencing analysis with four biological replicates per condition. Genes that are differentially expressed in worms supplemented with 500 μM glycine are highlighted in red. An adjusted *p*-value < 0.05 was set as the cut-off value for significantly differential expression.
(TIF)

S7 Fig Transcriptomic changes of glycine and one-carbon metabolism in *daf-2(e1370)*, *eat-2(ad465)*, and *mrps-5* RNAi worms. **(A-C)** Gene set map analyses performed on the KEGG gene sets including “glycine, serine and threonine metabolism” (cel00260), “cysteine and methionine metabolism” (cel100270), “one carbon pool by folate” (cel100670), and “purine metabolism” (cel100230) showing that these four KEGG gene sets are significantly upregulated with a *p*-value < 0.05 in *daf-2(e1370)* **(A)**, *eat-2 (ad465)* **(B)** (28), and *mrps-5* RNAi worms **(C)** compared to wild-type N2 respectively. Total RNA extracted from *daf-2(e1370)*, *eat-2(ad465)* (28), and *mrps-5* RNAi (reported here) from four biological replicates per condition, at young adult stage, were used for microarray analysis, respectively. Worms gene set map analyses were performed on “R2” platform and plotted in heat maps (z-score normalized). A *p*-value < 0.05 was used as the cut-off for differentially affected gene sets in every pair of comparison. **(D-F)** Heat maps showing that the majority of genes in “glycine, serine and threonine metabolism” from the KEGG gene sets are upregulated in *daf-2(e1370)* **(D)**, *eat-2(ad465)* **(E)** (28) and *mrps-5* RNAi worms (**F**) compared to N2 respectively (z-score normalized). **(G-I)** Heat maps showing that the majority of genes in “one carbon pool by folate” from the KEGG gene sets are upregulated in *daf-2(e1370)* **(G)**, *eat-2(ad465)* **(H)** (28), and *mrps-5* RNAi worms **(I).**
(TIF)

S8 Fig Gene expression of SAMe synthetase in long-lived worms. Heat map showing the expressions of *sams-1*, *sams-2*, *sams-3*, *sams-4*, and *sams-5* from the microarray data performed on long-lived worm models including *daf-2(e1370)*, *eat-2(ad465)* (28), and *mrps-5* RNAi.
(TIF)

S9 Fig Gene expression profile in purine metabolism in long-lived worms. **(A-C)** Genes in KEGG “purine metabolic pathway” in *C*. *elegans* were all included in the analysis. Previously reported microarray data of long-lived worms (28) was used to compare *daf-2(e1370)* **(A)**, *eat-2(ad465)* **(B),** and *mrps-5* RNAi worms **(C)** to N2 respectively for transcriptional changes, visualized in heat map (expression values were normalized by z-score transformation) (GEO accession number: GSE106672).
(TIF)

S10 Fig Genome-wide transcriptome comparison reveals that more genes are deactivated in *C. elegans* in response to serine treatment. **(A)** Differentially expressed genes in 5 mM serine-treated worms against 500 μM glycine-treated worms as showed in volcano plot. Grey dots indicate no differential regulation, red and blue dots indicate significant activation and repression based on adjusted *p*-values < 0.05 and log_2_-transformed fold change with absolute value > 0.5. Worm were fed UV-killed *E*. *coli* OP50 and supplemented with 500 μM glycine or 5 mM serine from the time of hatching. For total RNA extraction, worms were collected at adult D1 with four biological replicates per condition, then continued with RNA-sequencing analysis. **(B)** Differentially expressed genes in 5 mM serine compared to control displayed in volcano plot. Grey dots indicate no differential regulation, red and blue dots indicate significant activation and repression based on adjusted *p*-values < 0.05 and log_2_-transformed fold change with absolute value > 0.5.
(TIF)

S1 Table Statistical analysis of lifespan. Summary of median lifespan and statistical analysis (*p*-values) for lifespan experiments including different treatments and bacterial conditions displayed in **Figs 2B and 2D–2F**, **5A–5C, 6A–6D**, **S3**, **S4B Figs**. Larval stage 4 (L4) is considered as day 0 of the lifespan assay. The median lifespan and *p*-values were calculated by a log-rank (Mantel-Cox) statistical test. *P*-values less than 0.05 are considered statistically significant, demonstrating that the two lifespan populations are different. Cumulative statistics and statistics of individual experiments are shown for each condition. The total number of individuals scored, and independent experiments are shown.
(DOCX)

S2 Table List of primers used for qPCR in *C. elegans*. (DOCX)

S3 Table RNA-seq dataset. (XLSX)
