# Supplementary figures and images for: Glycine promotes longevity in Caenorhabditis elegans in a methionine cycle-dependent fashion
Source: PLoS Genet. 2019 Mar 7;15(3):e1007633. doi: 10.1371/journal.pgen.1007633 (PMC6424468; doi:10.1371/journal.pgen.1007633)

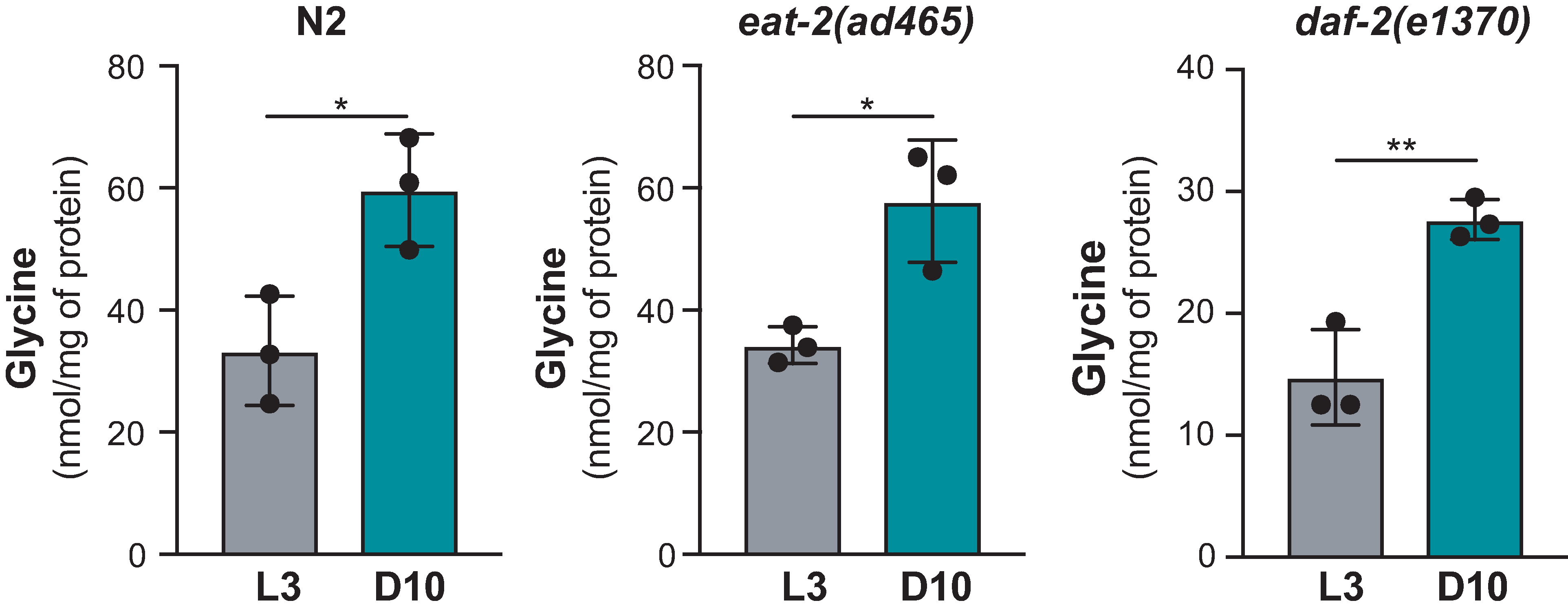

Supplement: S1 Fig — The levels of glycine were quantified by UPLC-MS/MS in young (L3) and aged (D10) wild type N2, daf-2(e1370) and eat-2(ad465) animals. The level of glycine is significantly higher at the age of D10 relative to the level of glycine at L3 in N2, daf-2(e1370) and eat-2(ad465). Worms were cultured on live E. coli OP50 and collected at the desired stage for amino acids extraction. Bar graphs are expressed as mean ± SD with three biological replicates; Significance was calculated using Student’s t-test; *p < 0.05, **p < 0.01. (TIF) [file pgen.1007633.s001.tif]

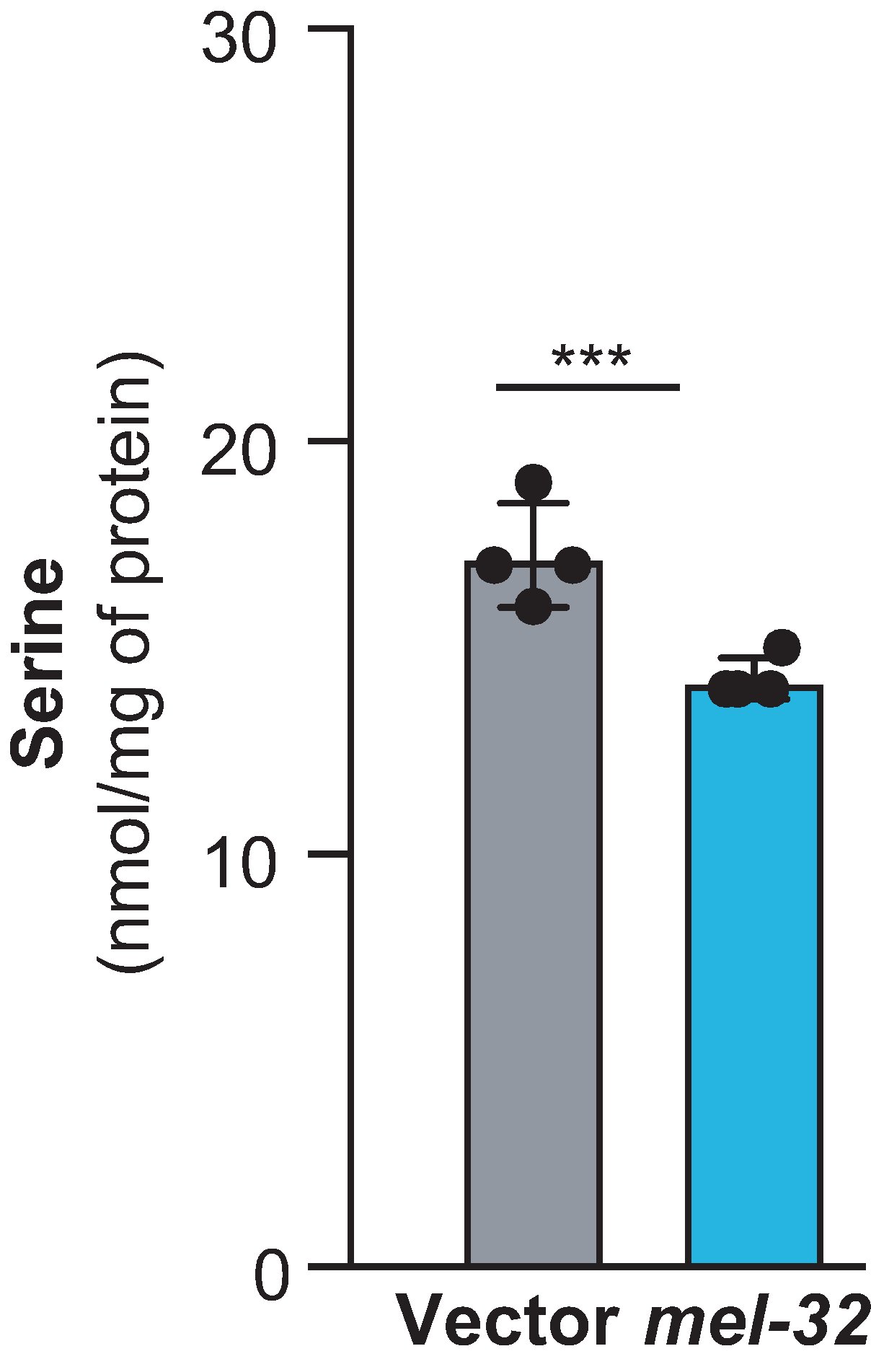

Supplement: S2 Fig — The level of serine measured by UPLC-MS/MS in worms subjected to RNAi against mel-32. Worms were fed HT115 RNAi bacteria against mel-32 from the time of hatching and collected for amino acids extraction at D1. Bar graphs are expressed as mean ± SD with four biological replicates; Significance was calculated using Student’s t-test; ***p < 0.001. (TIF) [file pgen.1007633.s002.tif]

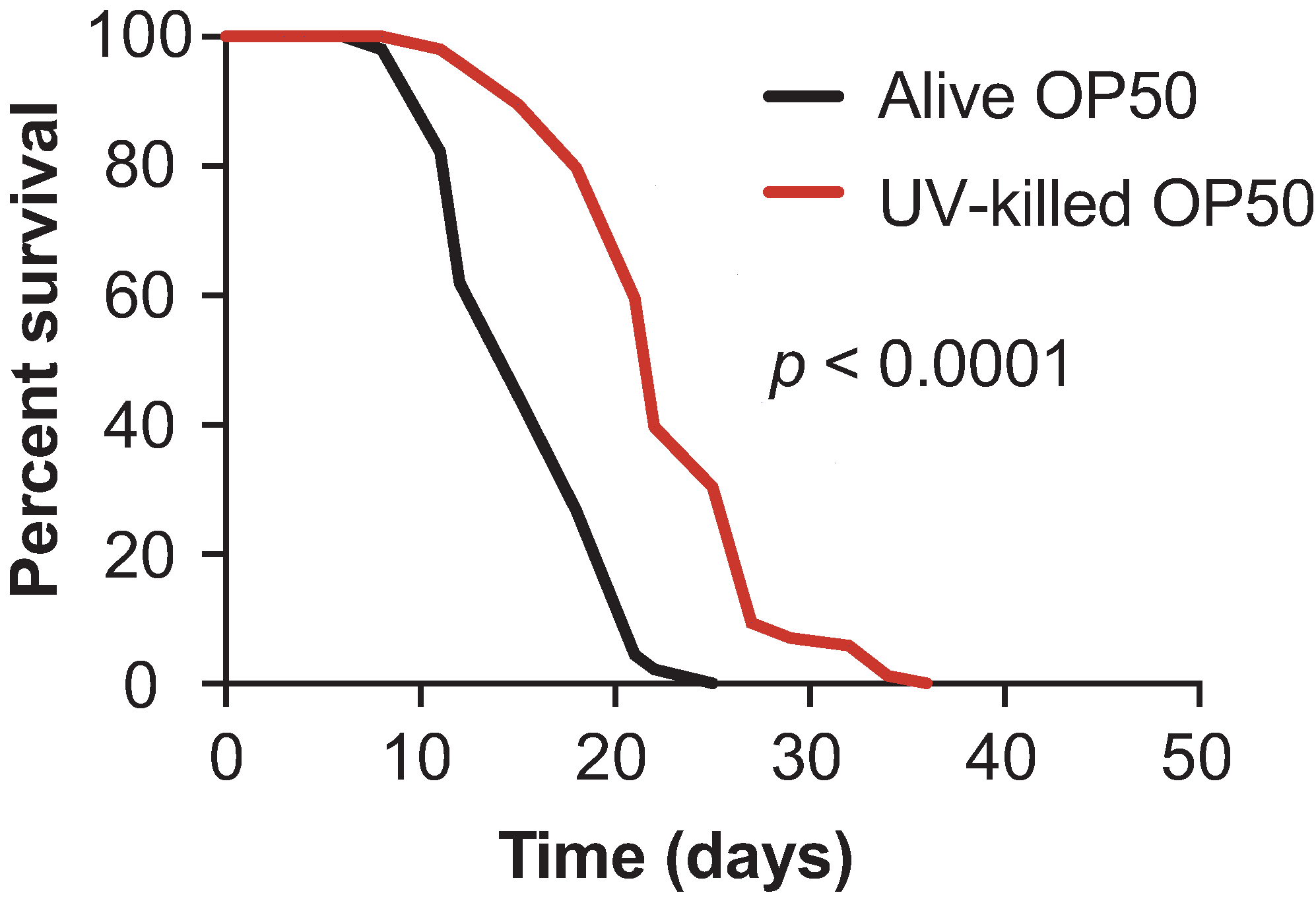

Supplement: S3 Fig — Lifespan analysis of C. elegans cultured on alive and UV-killed E. coli OP50, showing that the latter lives longer. Comparisons of survival curves was performed by log-rank tests. See S1 Table for lifespan statistics. (TIF) [file pgen.1007633.s003.tif]

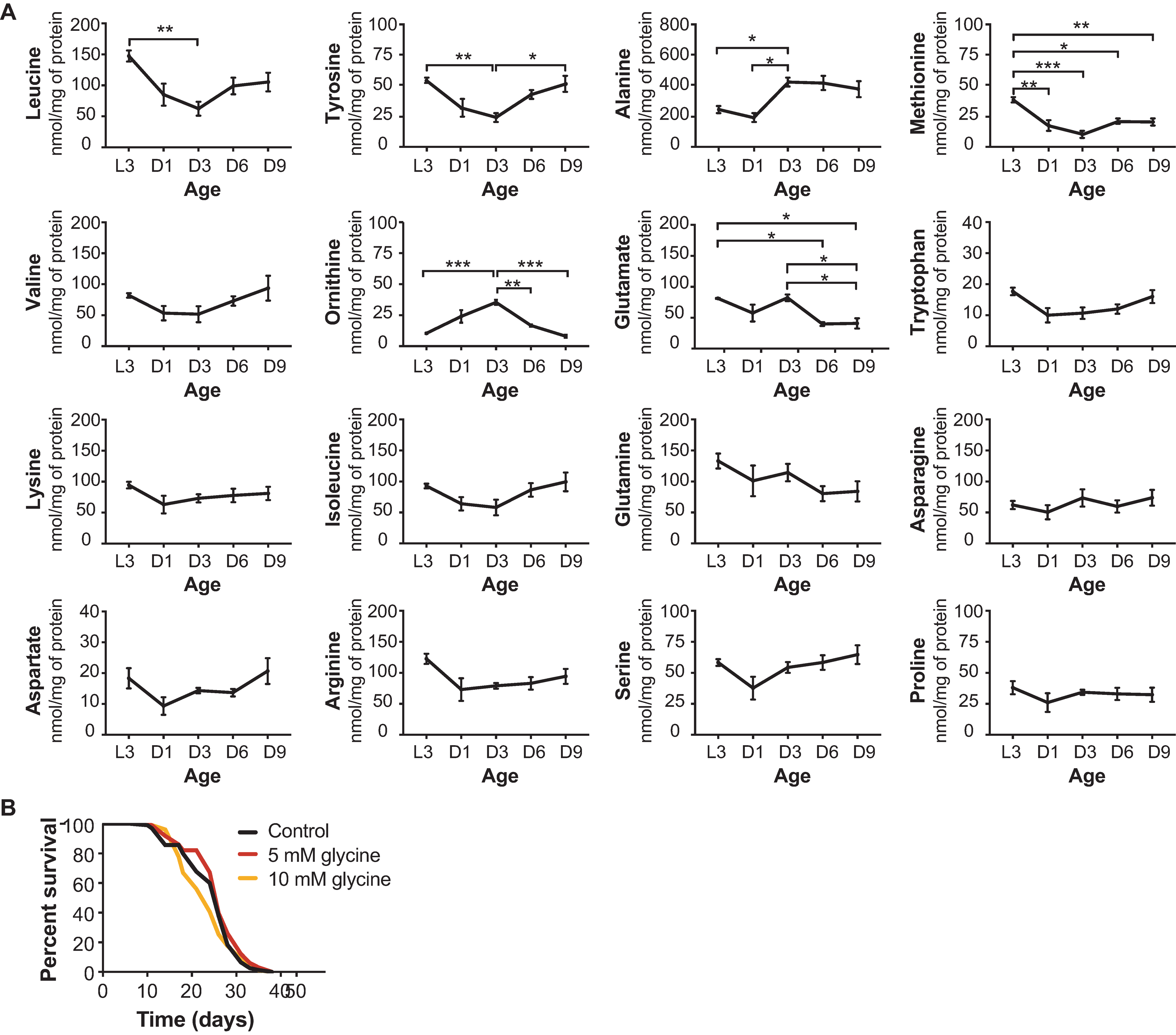

Supplement: S4 Fig — (A) Amino acid amounts were measured using UPLC-MS/MS across 5 time-points of the lifespan of worms fed UV-killed E. coli OP50 with three biological replicates, including L3, D1, D3, D6, and D9. Note: the profile of glycine is shown in Fig 2A. Statistical analysis between groups was performed using one-way ANOVA. Significance levels are indicated with asterisks as follows: *p < 0.05; **p < 0.01, ***p < 0.001. (B) Lifespan analyses of C. elegans cultured on UV-killed E. coli OP50 upon 5 mM and 10 mM glycine treatments showing that glycine supplementation at 5 mM and 10 mM has no effect on lifespan. Comparisons of survival curves was performed by log-rank tests. See S1 Table for lifespan statistics. (TIF) [file pgen.1007633.s004.tif]

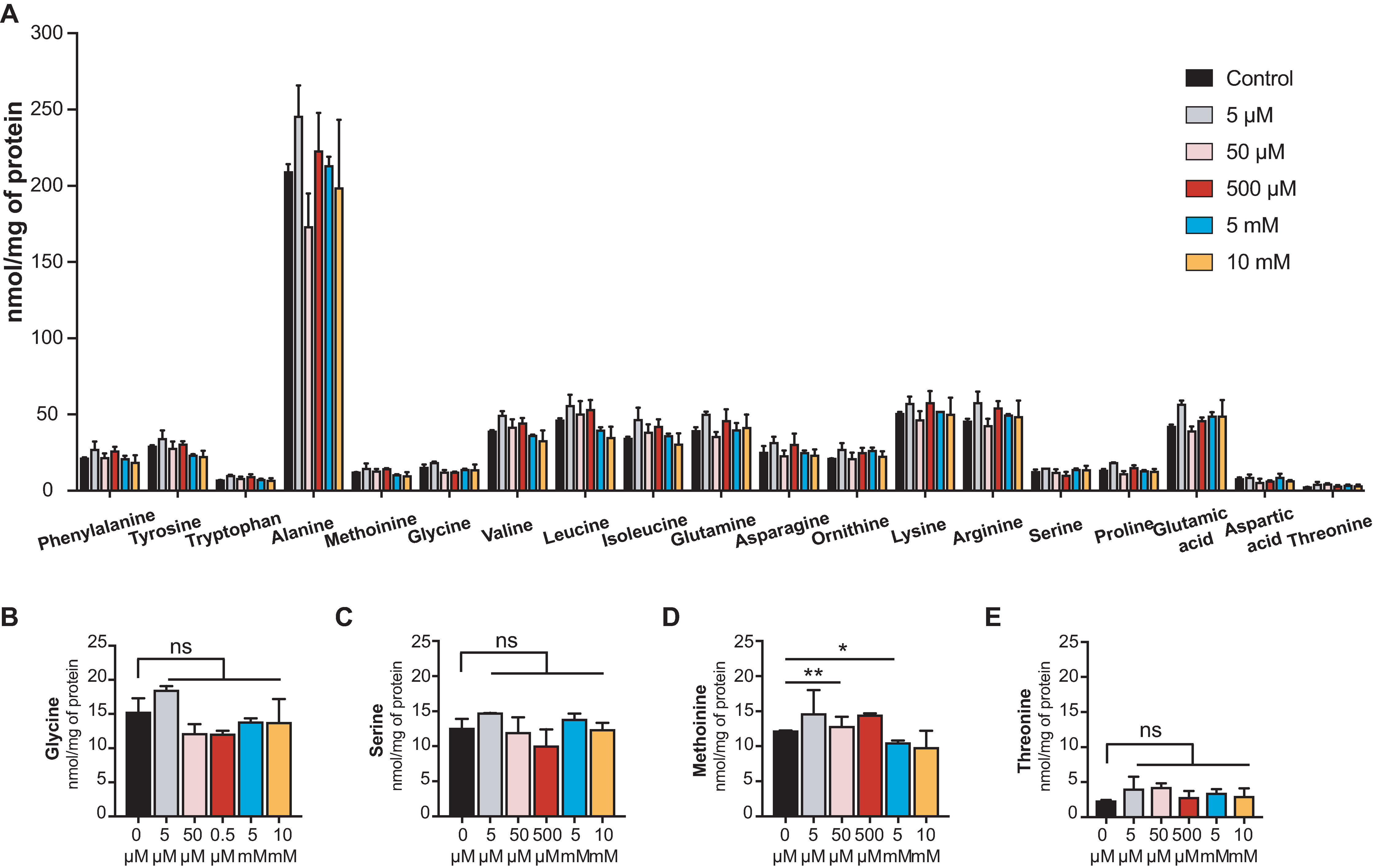

Supplement: S5 Fig — (A-D) Amino acid profile of D1 adult worms supplemented with increasing amounts of glycine ranging from 5 μM to 10 mM and fed UV-killed E. coli OP50. The levels of glycine, serine, methionine and threonine in (A) are presented in separate graphs (B), (C), (D), and (E) with the levels of significance indicated. Bar graphs are expressed as mean ± SD with three biological replicates; Statistical analysis was performed using one-way ANOVA. Significance levels are indicated with asterisks as follows: ns, not significant; *p < 0.05; **p < 0.01. (TIF) [file pgen.1007633.s005.tif]

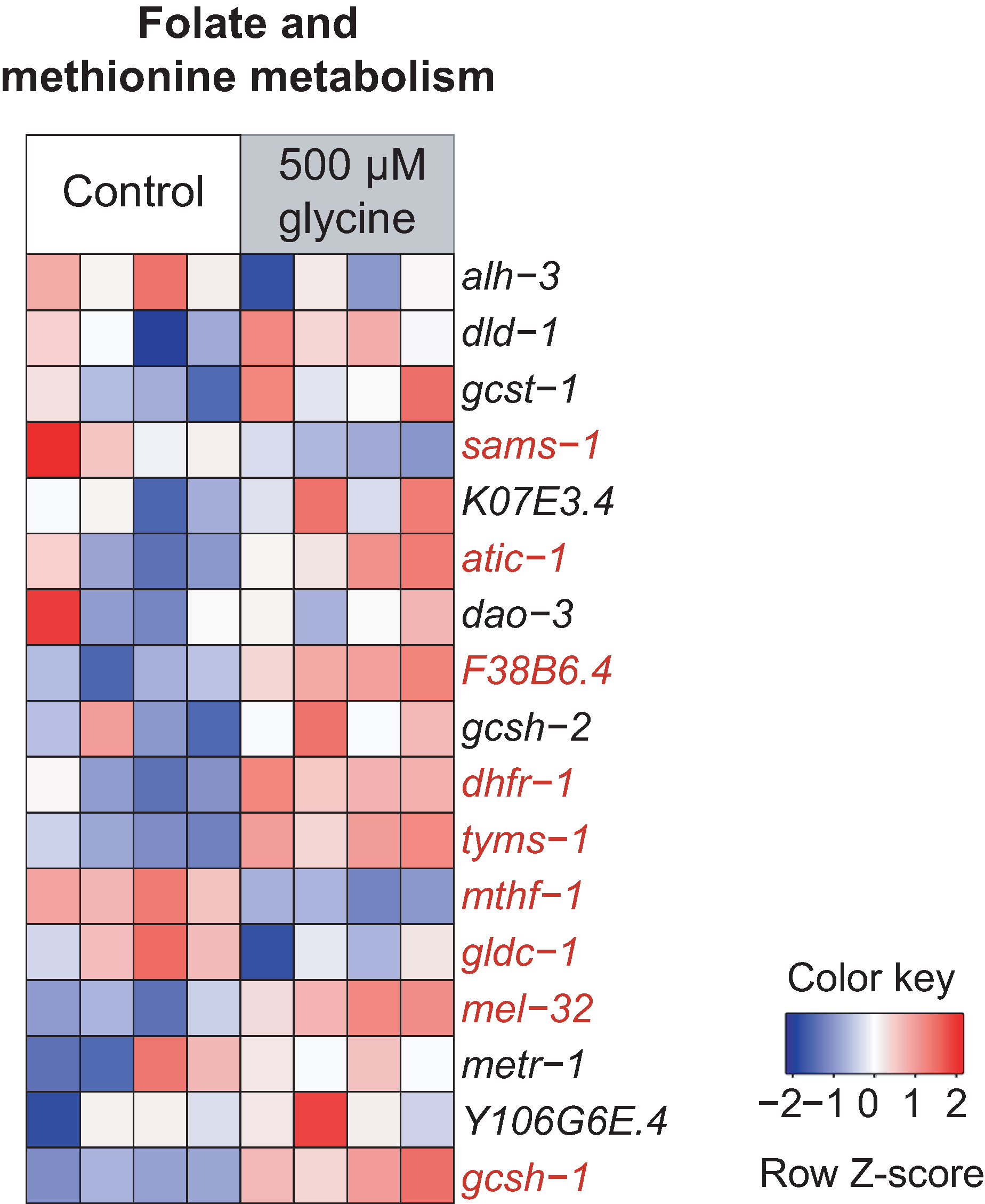

Supplement: S6 Fig — Heat map showing transcriptional changes of the genes in the Fig 3A diagram upon supplementation with 500 μM glycine (z-score normalized). Worms were fed UV-killed E. coli OP50 upon 500 μM glycine treatment from the time of hatching, and collected at adult D1 for total RNA extraction, then continued with RNA-sequencing analysis with four biological replicates per condition. Genes that are differentially expressed in worms supplemented with 500 μM glycine are highlighted in red. An adjusted p-value < 0.05 was set as the cut-off value for significantly differential expression. (TIF) [file pgen.1007633.s006.tif]

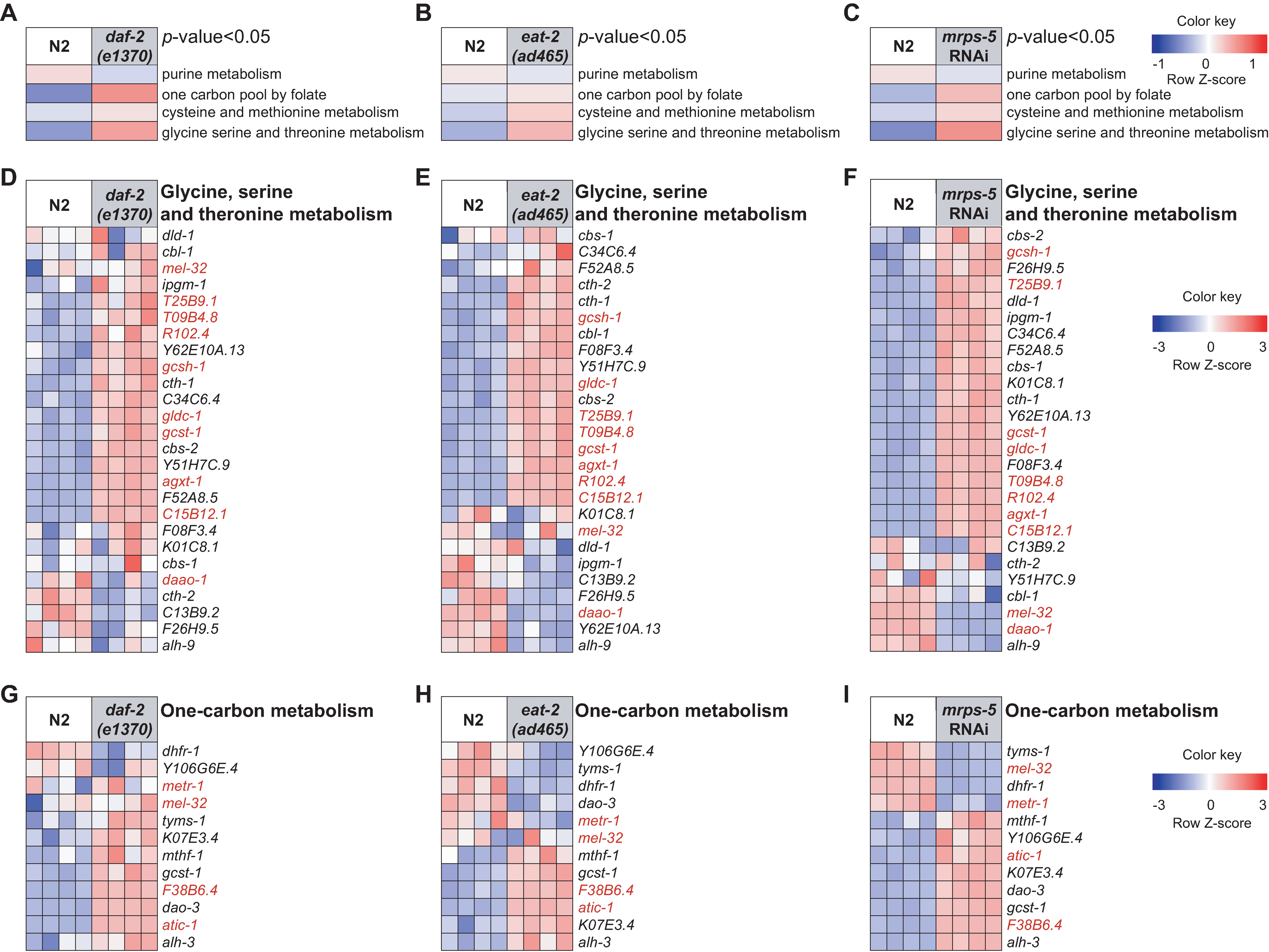

Supplement: S7 Fig — (A-C) Gene set map analyses performed on the KEGG gene sets including “glycine, serine and threonine metabolism” (cel00260), “cysteine and methionine metabolism” (cel100270), “one carbon pool by folate” (cel100670), and “purine metabolism” (cel100230) showing that these four KEGG gene sets are significantly upregulated with a p-value < 0.05 in daf-2(e1370) (A), eat-2 (ad465) (B) (28), and mrps-5 RNAi worms (C) compared to wild-type N2 respectively. Total RNA extracted from daf-2(e1370), eat-2(ad465) (28), and mrps-5 RNAi (reported here) from four biological replicates per condition, at young adult stage, were used for microarray analysis, respectively. Worms gene set map analyses were performed on “R2” platform and plotted in heat maps (z-score normalized). A p-value < 0.05 was used as the cut-off for differentially affected gene sets in every pair of comparison. (D-F) Heat maps showing that the majority of genes in “glycine, serine and threonine metabolism” from the KEGG gene sets are upregulated in daf-2(e1370) (D), eat-2(ad465) (E) (28) and mrps-5 RNAi worms (F) compared to N2 respectively (z-score normalized). (G-I) Heat maps showing that the majority of genes in “one carbon pool by folate” from the KEGG gene sets are upregulated in daf-2(e1370) (G), eat-2(ad465) (H) (28), and mrps-5 RNAi worms (I). (TIF) [file pgen.1007633.s007.tif]

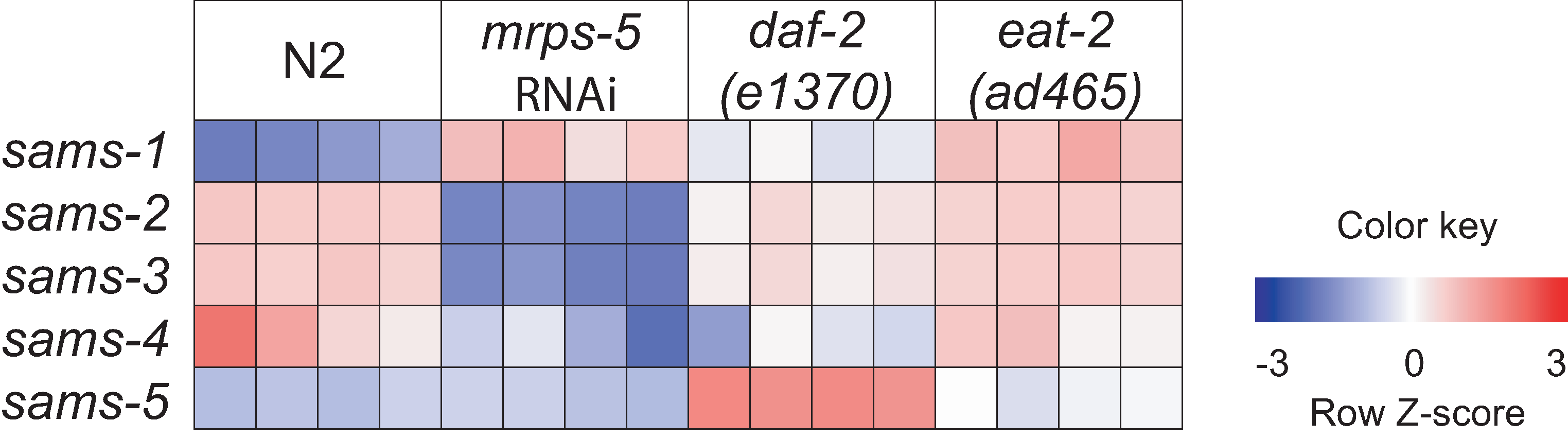

Supplement: S8 Fig — Heat map showing the expressions of sams-1, sams-2, sams-3, sams-4, and sams-5 from the microarray data performed on long-lived worm models including daf-2(e1370), eat-2(ad465) (28), and mrps-5 RNAi. (TIF) [file pgen.1007633.s008.tif]

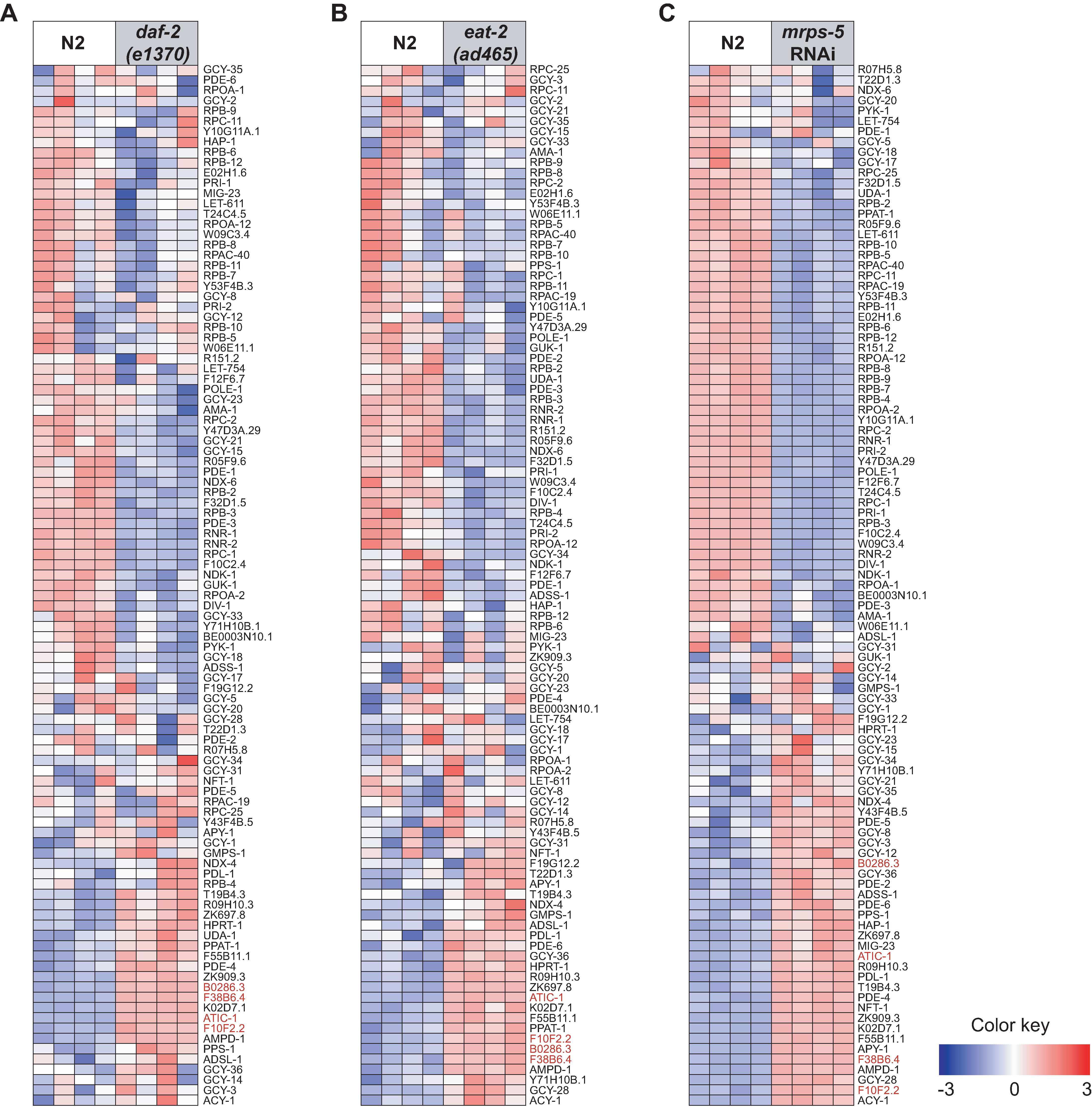

Supplement: S9 Fig — (A-C) Genes in KEGG “purine metabolic pathway” in C. elegans were all included in the analysis. Previously reported microarray data of long-lived worms (28) was used to compare daf-2(e1370) (A), eat-2(ad465) (B), and mrps-5 RNAi worms (C) to N2 respectively for transcriptional changes, visualized in heat map (expression values were normalized by z-score transformation) (GEO accession number: GSE106672). (TIF) [file pgen.1007633.s009.tif]

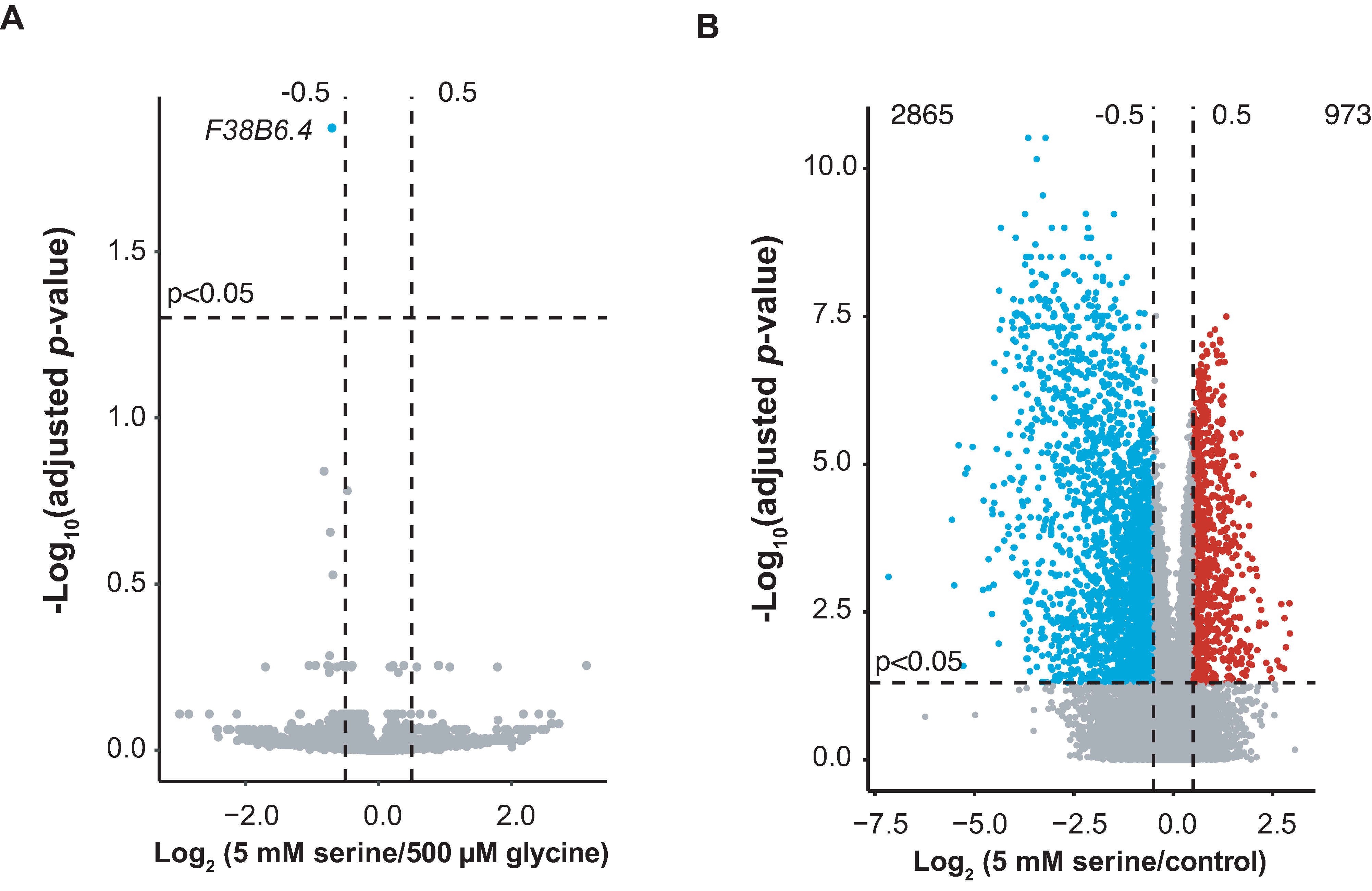

Supplement: S10 Fig — (A) Differentially expressed genes in 5 mM serine-treated worms against 500 μM glycine-treated worms as showed in volcano plot. Grey dots indicate no differential regulation, red and blue dots indicate significant activation and repression based on adjusted p-values < 0.05 and log2-transformed fold change with absolute value > 0.5. Worm were fed UV-killed E. coli OP50 and supplemented with 500 μM glycine or 5 mM serine from the time of hatching. For total RNA extraction, worms were collected at adult D1 with four biological replicates per condition, then continued with RNA-sequencing analysis. (B) Differentially expressed genes in 5 mM serine compared to control displayed in volcano plot. Grey dots indicate no differential regulation, red and blue dots indicate significant activation and repression based on adjusted p-values < 0.05 and log2-transformed fold change with absolute value > 0.5. (TIF) [file pgen.1007633.s010.tif]
