# Supplementary material for: Glycine promotes longevity in Caenorhabditis elegans in a methionine cycle-dependent fashion
Source: PLoS Genet. 2019 Mar 7;15(3):e1007633. doi: 10.1371/journal.pgen.1007633 (PMC6424468; doi:10.1371/journal.pgen.1007633)
Supplement: S1 Table — Summary of median lifespan and statistical analysis (p-values) for lifespan experiments including different treatments and bacterial conditions displayed in Figs 2B and 2D–2F, 5A–5C, 6A–6D, S3, S4B Figs. Larval stage 4 (L4) is considered as day 0 of the lifespan assay. The median lifespan and p-values were calculated by a log-rank (Mantel-Cox) statistical test. P-values less than 0.05 are considered statistically significant, demonstrating that the two lifespan populations are different. Cumulative statistics and statistics of individual experiments are shown for each condition. The total number of individuals scored, and independent experiments are shown. (DOCX) [file pgen.1007633.s011.docx]

| **Cumulative statistics** | | | | | | | | | **Statistics of individuals expts.** | | | | |
| --- | --- | --- | --- | --- | --- | --- | --- | --- | --- | --- | --- | --- | --- |
| **Strains** | **Bacteria** | **Supplementation** | **No. of expts.** | **Median lifespan (days)**  **(treatment vs control)** | **Change in lifespan (%)** | **S.E.M.**  **(treatment vs control)** | **Control**  **No. of animals**  **(dead vs censored)** | **Treatment**  **No. of animals**  **(dead vs censored)** | **Median lifespan (days)**  **(treatment vs control)** | **Change in lifespan (%)** | ***p*-value** | **Control**  **No. of animals**  **(dead vs censored)** | **Treatment**  **No. of animals**  **(dead vs censored)** |
| N2 | OP50  UV-  killed | Glycine  500 µM  (treatment throughout entire  life) | 7 | 30/25 | +20.0 | 0.34/0.34 | 780  (570/210) | 780  (538/242) | 31/26 | +19.2 | <0.0001 | 120  (81/39) | 120  (73/47) |
|  |  |  |  |  |  |  |  |  | 31/25 | +24.0 | <0.0001 | 120  (97/23) | 120  (75/45) |
|  |  |  |  |  |  |  |  |  | 31/29 | +6.8 | <0.0001 | 120  (97/23) | 120  (101/19) |
|  |  |  |  |  |  |  |  |  | 31/28 | +10.7 | <0.0001 | 120  (89/31) | 120  (95/25) |
|  |  |  |  |  |  |  |  |  | 28/21 | +33.3 | <0.0001 | 100  (64/36) | 100  (56/44) |
|  |  |  |  |  |  |  |  |  | 23/21 | +9.5 | <0.0001 | 100  (78/22) | 100  (72/28) |
|  |  |  |  |  |  |  |  |  | 28/21 | +33.3 | <0.0001 | 100  (64/36) | 100  (66/34) |
| N2 | OP50  UV-  killed | Glycine  500 µM  (treatment from egg to larval stage 4) | 3 | 21/21 | 0 | 0.49/0.38 | 300  (206/94) | 300  (166/134) | 23/21 | +9.5 | 0.184 | 100  (64/36) | 100  (54/46) |
|  |  |  |  |  |  |  |  |  | 21/21 | 0 | 0.953 | 100  (78/22) | 100  (69/31) |
|  |  |  |  |  |  |  |  |  | 21/21 | 0 | 0.371 | 100  (64/36) | 100  (43/57) |
| N2 | OP50  UV-  killed | Glycine  500 µM  (treatment from day1 to day3 of adulthood) | 3 | 25/21 | +19.0 | 0.70/0.37 | 300  (206/94) | 300  (191/109) | 25/21 | +19.0 | 0.0008 | 100  (64/36) | 100  (64/36) |
|  |  |  |  |  |  |  |  |  | 23/21 | +9.5 | <0.0001 | 100  (78/22) | 100  (70/30) |
|  |  |  |  |  |  |  |  |  | 30/21 | +42.9 | <0.0001 | 100  (64/36) | 100  (57/43) |
| N2 | OP50  UV-  killed | Glycine  500 µM  (treatment throughout adulthood only) | 3 | 25/21 | +19.0 | 0.79/0.37 | 300  (206/94) | 300  (164/136) | 28/21 | +33.3 | <0.0001 | 100  (64/36) | 100  (52/48) |
|  |  |  |  |  |  |  |  |  | 23/21 | +9.5 | <0.0001 | 100  (78/22) | 100  (56/44) |
|  |  |  |  |  |  |  |  |  | 28/21 | +33.3 | 0.0007 | 100  (64/36) | 100  (56/44) |
| N2 | OP50  UV-  killed | Glycine  5 µM | 2 | 28/26 | +7.7 | 0.72/0.42 | 240  (178/62) | 240  (158/82) | 28/26 | +7.7 | <0.0001 | 120  (81/39) | 120  (75/45) |
|  |  |  |  |  |  |  |  |  | 27/25 | +8.0 | 0.001 | 120  (97/23) | 120  (83/37) |
| N2 | OP50  UV-  killed | Glycine  50 µM | 2 | 31/26 | +19.2 | 0.48/0.42 | 240  (178/62) | 240  (164/76) | 31/26 | +19.2 | <0.0001 | 120  (81/39) | 120  (76/44) |
|  |  |  |  |  |  |  |  |  | 29/25 | +16.0 | <0.0001 | 120  (97/23) | 120  (88/32) |
| N2 | OP50  UV-  killed | Glycine  5 mM | 2 | 26/26 | 0 | 0.48/0.42 | 240  (178/62) | 240  (146/94) | 26/26 | 0 | 0.169 | 120  (81/39) | 120  (70/50) |
|  |  |  |  |  |  |  |  |  | 27/25 | +8.0 | 0.147 | 120  (97/23) | 120  (76/44) |
| N2 | OP50  UV-  killed | Glycine  10 mM | 2 | 24/26 | -7.7 | 0.54/0.42 | 240  (178/62) | 240  (174/66) | 24/26 | -7.7 | 0.283 | 120  (81/39) | 120  (92/28) |
|  |  |  |  |  |  |  |  |  | 25/25 | 0 | 0.852 | 120  (97/23) | 120  (82/38) |
| *metr-1*  *(ok521)* | OP50  UV-  killed | Glycine  500 µM | 2 | 21/21 | 0 | 0.23/0.21 | 300  (179/121) | 300  (149/151) | 21/21 | 0 | 0.899 | 120  (75/45) | 120  (59/61) |
|  |  |  |  |  |  |  |  |  | 21/21 | 0 | 0.665 | 180  (104/76) | 180  (90/90) |
| *sams-1*  *(ok3033)* | OP50  UV-  killed | Glycine  500 µM | 2 | 23/23 | 0 | 0.35/0.33 | 252  (166/86) | 235  (155/80) | 23/23 | 0 | 0.111 | 132  (87/45) | 120  (83/37) |
|  |  |  |  |  |  |  |  |  | 23/23 | 0 | 0.213 | 120  (79/41) | 115  (72/43) |
| N2 | OP50  UV-  killed | Serine  5 mM  (treatment throughout entire  life) | 5 | 28/22 | +27.3 | 0.49/0.37 | 564  (397/167) | 564  (396/168) | 29/24 | +20.8 | <0.0001 | 120  (73/47) | 120  (78/42) |
|  |  |  |  |  |  |  |  |  | 31/24 | +29.2 | <0.0001 | 144  (116/28) | 144  (94/50) |
|  |  |  |  |  |  |  |  |  | 25/21 | +19.0 | <0.0001 | 100  (64/36) | 100  (66/34) |
|  |  |  |  |  |  |  |  |  | 25/21 | +19.0 | <0.0001 | 100  (78/22) | 100  (75/25) |
|  |  |  |  |  |  |  |  |  | 28/21 | +33.3 | <0.0001 | 100  (64/36) | 100  (67/33) |
| N2 | OP50  UV-  killed | Serine  5 mM  (treatment from egg to larval stage 4) | 3 | 21/21 | 0 | 0.59/0.37 | 300  (206/94) | 300  (165/135) | 23/21 | +9.5 | 0.3241 | 100  (64/36) | 100  (45/55) |
|  |  |  |  |  |  |  |  |  | 18/21 | -14.3 | 0.4698 | 100  (78/22) | 100  (68/32) |
|  |  |  |  |  |  |  |  |  | 19/21 | -9.5 | 0.6297 | 100  (64/3) | 100  (52/48) |
| N2 | OP50  UV-  killed | Serine  5 mM  (treatment from day1 to day3 of adulthood) | 3 | 25/21 | +19.0 | 0.85/0.37 | 300  (206/94) | 300  (189/111) | 28/21 | +33.3 | <0.0001 | 100  (64/36) | 100  (60/40) |
|  |  |  |  |  |  |  |  |  | 21/21 | 0 | <0.01 | 100  (78/22) | 100  (68/32) |
|  |  |  |  |  |  |  |  |  | 30/21 | +42.9 | <0.0001 | 100  (64/36) | 100  (61/39) |
| N2 | OP50  UV-  killed | Serine  5 mM  (treatment throughout adulthood only) | 3 | 25/21 | +19.0 | 0.72/0.37 | 300  (206/94) | 300  (191/109) | 28/21 | +33.3 | <0.0001 | 100  (64/36) | 100  (61/39) |
|  |  |  |  |  |  |  |  |  | 23/21 | +9.5 | <0.0001 | 100  (78/22) | 100  (61/39) |
|  |  |  |  |  |  |  |  |  | 28/21 | +33.3 | <0.0001 | 100  (64/36) | 100  (69/31) |
| *metr-1*  *(ok521)* | OP50  UV-  killed | Serine  5 mM | 2 | 19/17 | +11.8 | 0.55/0.45 | 264  (167/97) | 264  (186/78) | 21/21 | 0 | 0.103 | 120  (68/52) | 120  (71/49) |
|  |  |  |  |  |  |  |  |  | 19/17 | +11.8 | 0.202 | 144  (99/45) | 144  (115/29) |
| *sams-1*  *(ok3033)* | OP50  UV-  killed | Serine  5 mM | 2 | 22/24 | -8.3 | 0.91/0.64 | 264  (185/79) | 264  (150/114) | 20/20 | 0 | 0.146 | 120  (67/53) | 120  (63/57) |
|  |  |  |  |  |  |  |  |  | 24/24 | 0 | 0.347 | 144  (118/26) | 144  (87/57) |
| N2 | HT115  (*gcst-1*) | - | 3 | 19/19 | 0 | 0.72/0.63 | 432  (358/74) | 432  (364/68) | 18/18 | 0 | 0.368 | 144  (122/22) | 144  (113/31) |
|  |  |  |  |  |  |  |  |  | 19/19 | 0 | 0.459 | 144  (113/31) | 144  (129/15) |
|  |  |  |  |  |  |  |  |  | 20/18 | +11.1 | 0.553 | 144  (123/21) | 144  (122/22) |
| N2 | HT115  (*mel-32*) | - | 3 | 20/18 | +11.1 | 0.34/0.24 | 408  (315/93) | 408  (340/68) | 21/19 | +10.5 | <0.0001 | 120  (76/44) | 120  (92/28) |
|  |  |  |  |  |  |  |  |  | 18/18 | 0 | <0.001 | 144  (126/18) | 144  (127/17) |
|  |  |  |  |  |  |  |  |  | 20/18 | +11.1 | <0.0001 | 144  (113/31) | 144  (121/23) |
