# Supplementary material for: Glycine promotes longevity in Caenorhabditis elegans in a methionine cycle-dependent fashion
Source: PLoS Genet. 2019 Mar 7;15(3):e1007633. doi: 10.1371/journal.pgen.1007633 (PMC6424468; doi:10.1371/journal.pgen.1007633)
Supplement: S2 Table — (DOCX) [file pgen.1007633.s012.docx]

| **Gene symbol** | **Gene ID** | **Forward** | **Reverse** |
| --- | --- | --- | --- |
| *R102.4* | 177961 | GGCGAGGAGATAATCGTCGG | GTGACAATCGGGTATACTCGTCA |
| *mel-32* | 175915 | AACCGGAGGAACCGACAATC | TTTGCGATTTGAACACCCTCA |
| *T09B4.8* | 172301 | GTCATCCAAAAATCAACGCAGC | ACGAGCAAGAGCTAAAGCCA |
| *C15B12.1* | 180921 | AGCACAACGATCACAACGAT | ACGGTTGTCTTTTTGCCAGC |
| *gldc-1* | 174012 | GTCGGGCGTGTTGCTTTTC | TTTGGCACATTGGTTCCGGT |
| *F38B6.4* | 180935 | GCAAGGGAGCATGCAAACAA | CAAAACCCCCAATCTGTGCC |
| *daao-1* | 3565775 | ATTACGCGCTGTTGAACGAC | GGATCCACCGTGCCCATAAT |
| *gcst-1* | 178960 | GCTGAAGCGTCTGCAAAACA | GTCAGCGTACTGAGTTGGCA |
| *dld-1* | 178387 | TCGCCGAAGCCACGTTGGCAAT | CCGAGAGGGTTGGATGTGGATGGC |
| *gcsh-1* | 183902 | GAGCGGTGGAGAGTGTCAAGGCAG | AAGCCAACCCTTCTCCAGTGGGC |
| *mthf-1* | 174254 | TCGATTCTCTGTCCACAATGC | TCGATTCTCTGTCCACAATGC |
| *metr-1* | 174681 | GTGGAGAGATCCTCAAAGACCA | CTCCGGCTTCCAAGTAGAGC |
| *sams-1* | 181370 | CCAGCATTGGATTCGACCAC | TCCGACATCTTCTCCGTCCT |
| *dhfr-1* | 172681 | TGGCAGGACGGCTAAACATT | CGCTTCGAGCGAGTTTACAA |
| *tyms-1* | 172149 | GACAAATGGTTCTGCCACCG | TCCGAGACCCATATCTCCACT |
| *agxt-1* | 174414 | GCTCCGCAGGGGCAAGCAGT | CCACCGAGGGACGCCACCGTA |
| *T25B9.1* | 177968 | AGCAGAAGATTCTCGATTCCG | GACCTTCAACAATGCGTCGG |
| *gss-1* | 174493 | GCTGTTGAATGCTCCAAGGC | AAGCCCATTAGCATGAGCCC |
| *Y45F10D.4* | 178344 | GTCGCTTCAAATCAGTTCAGC | GTTCTTGTCAAGTGATCCGACA |
| *F35G12.2* | 175598 | ACTGCGTTCATCCGTGCCGC | TGCGGTCCTCGAGCTCCTTC |
| *eif-3.C* | 172858 | GCTCGTGGAAAACGAACGAC | CTCTTGAGTGTCATCCTTCAGA |
